# Single-cell mRNA analysis and surface marker expression profiling of circulating immune cells in humans with alpha-gal syndrome

**DOI:** 10.3389/fimmu.2025.1629310

**Published:** 2025-09-30

**Authors:** Shailesh K. Choudhary, Scott P. Commins

**Affiliations:** ^1^ Thurston Research Center, Division of Allergy, Immunology and Rheumatology, Department of Medicine, University of North Carolina, Chapel Hill, NC, United States; ^2^ UNC Food Allergy Initiative, Department of Pediatrics, University of North Carolina, Chapel Hill, NC, United States; ^3^ Institute for Global Health and Infectious Diseases, University of North Carolina, Chapel Hill, NC, United States

**Keywords:** alpha-gal, alpha-gal syndrome (AGS), *Amblyomma americanum*, BD Rhapsody, red meat allergy, single-cell RNA-Seq, food allergy, lone star tick

## Abstract

**Introduction:**

Alpha-gal syndrome (AGS) is an IgE-mediated allergy to the oligosaccharide galactose-alpha-1,3-galactose (alpha-gal). Alpha-gal is found in the tissues of non-catarrhine mammals, and the characteristic delayed reactions are caused by the consumption of red meat, visceral organs, dairy, gelatin, and other products, including medications sourced from non-primate mammals. Although the syndrome is not nationally notifiable, it is estimated that 450,000 cases exist, making AGS the tenth-most common food allergy in the US. The syndrome is profoundly influenced by geographic locale, reflecting the important role of tick bites in sensitization. However, the specific immune cells, their interactions, and the downstream signaling cascades triggered by tick bites are not well understood. To address this gap, we conducted a comprehensive study to analyze the immune cells in the blood of AGS subjects compared to those of healthy controls.

**Methods:**

Peripheral blood mononuclear cell preparations were enriched for B cells from the same patient and sequentially labeled with sample tags and BD AbSeq oligo-conjugated antibodies. Sequencing libraries were prepared to targeted mRNA, antibody-oligonucleotides, and sample tags from AGS and control subjects. Multimodal analysis of both transcriptomes and surface marker expression of immune cells at the single-cell level was used to profile the immune response in AGS.

**Results:**

Several clusters of cells were unique to AGS subjects, including natural killer B (NKB) cells, natural killer T (NKT) cells and most notably, a circulating mast cell progenitor population. In addition, subjects with AGS had increased expression of several genes involved in immunomodulation and type 2 immunity. Although rare, we identified and characterized alpha-gal-specific memory B cells and alpha-gal-specific IgE-secreting cells. Our findings also revealed the presence of IgE-secreting transcripts, along with other classes of immunoglobulins (Ig), in a single cell, suggesting a unique pattern of Ig gene arrangements and class switching in B cells.

**Conclusions:**

Tick bites appear to induce a population of circulating mast cell progenitors and innate-like cells, such as NKT and NKB cells, that may play a critical role in sensitization to alpha-gal. Furthermore, alpha-gal-specific IgE is secreted by a heterogeneous population of B cells, including CCR6-proficient B cells and CCR6-deficient plasmablast/plasma cells.

## Introduction

Alpha-gal syndrome (AGS) is an allergic condition characterized by an immunoglobulin E (IgE)-mediated response to galactose-alpha-1,3-galactose (alpha-gal) (1, 2). Alpha-gal is an oligosaccharide found on glycolipids and glycoproteins in most non-primate mammalian meat and derived products (3). The allergic response to alpha-gal is unique: it appears to be induced by a tick bite, can occur after years of safely tolerating beef, pork, or lamb, and consumption of red meat typically causes a delayed-onset reaction 2–6 hours after ingestion (4). Parenteral administration, however, causes an immediate-onset allergic reaction, as was reported with cetuximab, a chimeric (mouse/human) monoclonal antibody heavily glycosylated with alpha-gal (4–6). The delay in triggering anaphylaxis appears to be due to antigen processing, likely associated with slower release of glycolipids into the bloodstream (7). In keeping with this, patients report more consistent and more severe reactions to fatty meat – underscoring the likely involvement of alpha-gal-containing glycolipids in allergic reactions (8).

Since the discovery of AGS in 2009, the number of positive tests for alpha-gal sIgE has been steadily increasing. Between 2010 and 2022, over 110,000 suspected cases of AGS were identified, with the total number of suspected cases of AGS estimated at 450,000 (9, 10). Most of these cases were reported in counties located within the southern, midwestern, and mid-Atlantic U.S. Census Bureau regions, which overlap with the geographical range of *Amblyomma americanum* (*A. americanum*), also known as the lone star tick (9, 10). In a recent case-control study, tick bites were formally identified as a risk factor for AGS (11). Along with epidemiological evidence, prospective studies have shown that after being bitten by *A. americanum* nymphs, there is a simultaneous increase in total and alpha-gal specific IgE levels and a decrease in those same values in the absence of ongoing exposure (12, 13). We have recently demonstrated that a nymphal bite from *A. americanum*, but not *Amblyomma maculatum*, induces AGS in mice deficient in alpha-gal (14). There are other reports demonstrating that cutaneous inoculation of *A. americanum* extract led to alpha-gal sIgE production in a mouse model (15, 16). Further, immunotherapy with alpha-gal glycolipid-containing nanoparticles in a mouse model blunts the production of Th-2 cytokines and alpha-gal sIgE (17). In keeping with tick bite as a risk factor for AGS, bites from *Ixodes ricinus*, *Ixodes holocyclus*, *Amblyomma sculptum*, and *Haemaphysalis longicornis* have each been associated with red meat allergy in Europe, Australia, Brazil, and Japan, respectively (18–23). Interestingly, ticks associated with AGS contain alpha-gal in their saliva or gut, suggesting that, along with the immunomodulatory activity of tick saliva, the presence of alpha-gal antigen in tick saliva may be critical to the development of alpha-gal IgE (24, 25). The recent discovery of alpha-gal glycolipids in the saliva of *A. americanum* and demonstration that the salivary gland extract could activate basophils from AGS subjects suggests that this pathway is mechanistically and immunologically plausible (26, 27). It is well documented that tick infestation of a vertebrate host delays wound healing, suppresses pro-inflammatory cytokines, and favors Th2 cytokines (by limiting Th1 responses in an attempt to evade the host cytotoxic T cell response) (28). Despite these insights, the immunologic events following tick bites that lead to the production of alpha-gal sIgE remain poorly understood.

In this study, we utilized the BD Rhapsody platform to perform both single-cell RNA-sequencing (scRNA-seq) and simultaneously profile cell surface marker expression to comprehensively characterize the distinct immune cell populations associated with AGS. We found unique clusters of immune cells in AGS subjects, with notable differences in cell transcriptomes, particularly in B cells, plasmablast (PB)/plasma cells (PC), natural killer (NK) cells, natural killer T (NKT) cells, natural killer B (NKB) cells, and monocytes. Furthermore, we were able to identify and characterize B cell subsets that produced alpha-gal sIgE. Additionally, we observed the rare presence of mast cell progenitors in the peripheral blood mononuclear cells (PBMCs) of participants with AGS.

## Materials and methods

### Reagents

BD Human Single Cell Multiplexing Kit (#633781), BD AbSeq Antibody-oligos (AbSeq Ab-O), BD Rhapsody BD Rhapsody Cartridge Kit (#633733), BD Rhapsody Cartridge Reagent Kit (#633731), BD Rhapsody cDNA Kit (#633774), BD Rhapsody Targeted mRNA and AbSeq Amplification Kit (#633774) and BD Rhapsody Immune Response Panel (human) were all obtained from BD Bioscience (Milpitas, CA). Seventeen human AbSeq Ab-O were used in this experiment: CD3 clone SK7 (#940000), CD4 clone SK3 (#940001), CD14 (#940005), CD56 clone NCAM16.2 (#940007), CD25 clone 2A3 (#940009), CD38 clone HIT2 (#940013), CD20 (#940016), CD27 clone M-T271 (#940018), CD123 clone7G3 (#940020), IgD clone IA6-2 (#940026), IgG (#940027), CD24 clone ML5 (#940028), CD196 (CCR6) clone 11A9 (#940033), CD40 clone 5C3 (#940049), CD19 clone SJ25C1 (#940004), IGM clone G20-127 (#940276) and custom conjugated Cetuximab Ab-O. BD Rhapsody Immune Response Panel (**Supplementary Table S1**) was supplemented with a custom gene panel constructed by BD and included *CCR6*, *Cd300c*, *Cd300lb*, *Cd300lf, Cd40lg, Flg, Gata3, Il9r, Mapk1, Mapk3, Mapk8, Mc1r, Mc3r, Mc5r, Pten, Spi1, Spink5, Tlr4, Tnfrsf1a* and *Tslp*. Besides the BD Rhapsody Kit index primers, additional TruSeq combinatorial dual index primers were obtained through IDT (see **Supplementary Table S2**).

### Isolation of PBMCs and enrichment of B cells

Blood was collected into acid citrated-dextrose tubes and PBMCs were isolated by Ficoll gradient (Ficoll-Paque Plus, Cytivia, Marlborough, MA) centrifugation as per the manufacturer’s instructions. PBMCs were incubated with RBC lysis buffer (Thermo Fisher Scientific, Waltham, MA) to remove traces of RBCs and then subjected to B cell purification involving negative selection (STEMCELL Technologies, Vancouver, Canada) according to the manufacturer’s protocol.

### Single-cell labeling, targeted transcriptome, and protein library preparation and sequencing

Half a million PBMCs were spiked with half a million enriched B cells from the same subject and labeled with sample tags using the BD Human Single-Cell Multiplexing Kit. Each tagged sample was washed twice with FACS staining buffer and counted. Tagged cells from two donors, a million each, were pooled and subjected to a sequential labeling workflow with BD AbSeq Ab-Oligos according to the manufacturer’s protocol. Briefly, non-specific Fc receptors on cells were blocked with human BD Fc Block, cells were washed twice and resuspended in 100 μL of FACS staining buffer before adding 100 μL 2X BD AbSeq labeling master mix. The pooled sample was washed twice, counted, and resuspended in a cold BD Sample Buffer to achieve approximately 40,000 captured cells. Single cells were then captured, and cDNA was synthesized, followed by targeted amplification of transcripts using the Human Immune Response Panel primers (399 genes) and a custom supplemental panel set (20 targets), as well as amplification of cDNA of sample Tag and AbSeq (17 parameters). Final RNA-seq libraries were prepared by performing the index PCR using a combination of 3 forward index primers (TreSeq D501-D503) and 12 reverse index primers (TrueSeq D701-D712). The quality of the final libraries was evaluated using the Agilent 2200 TapeStation, and quantification was done using the Qubit Fluorometer and Qubit dsDNA HS Kit. Final libraries were diluted to 4 nM, all libraries were pooled, spiked with 20% PhiX, and subjected to paired sequencing (custom length 60 X 8 X 42) on a Novaseq 6000 S4 XP platform (Illumina, San Diego, CA). We followed BD guidelines for sequencing depth for different libraries: 8000 reads/cell for the RNA-targeted library, 600 reads/cell for the sample tag library, and 500 reads/cell/AbSeq for the AbSeq library.

### Processing of transcriptomics data on the seven bridges platform

Transcriptomics data obtained in the FASTQ files were processed via the standard Rhapsody analysis pipeline (BD Biosciences, Milpitas, CA) on the Seven Bridges Platform (https://www.sevenbridges.com). Data obtained from the Rhapsody pipeline was imported into SeqGeq v1.8 and subjected to quality control to remove cells that were not singlets or if the sample tag was not uniquely determined. A natural log normalization using a scale factor of 10,000 was performed across the library for each cell, and thereafter, the Seurat plug-in was utilized to perform a ‘weighted-nearest neighbor’ (WNN) multimodal single-cell analysis, which utilizes both transcriptomes as well as cell-surface proteins (AbSeq parameters) from the same cell selected on the classification model huPBMC and DE stat model negative binomial. The WNN workflow comprises three main steps: (i) independently transforming and reducing the dimensionality of each modality, (ii) learning the modality ‘weight’ to construct WNN graphs, then integrating the modalities, and (iii) visualizing with UMAP (uniform manifold approximation and projection) and graph-based clustering (29). The plug-in output data includes a QC plot, modality weights, RNA heatmap, antibody-derived tag (ADT) heatmap, UMAP (WNN, RNA, and ADT), and a list of upregulated and downregulated genes in each cluster. Differential gene expression was analyzed in control vs. alpha-gal allergic participants in each cluster using the volcano plot in SeqGeq. The ViolinBox plug-in was used for data visualization and to create a heatmap.

## Results

### Characterization of circulating immune cells based on surface labeling with AbSeq Ab-Oligos

We utilized a multiomics approach for scRNA-seq, including surface labeling and targeted transcriptomes, from PBMCs of 18 alpha-gal allergic and 10 control subjects to characterize the cell types and gene expression associated with AGS (**Table 1**). Participants with AGS were confirmed to be allergic based on a history of reactions or reaction upon observed food challenge; however, samples were collected in the absence of ongoing allergic reactions or symptoms. The participants were at ‘resting’ state, practicing an appropriate avoidance diet, in the absence of symptoms.

**Table 1:** Characteristics of alpha-gal-allergic (AGS) and control subjects.

UNC_ID	Sex	Age	Allergy status	‡*sIgE	‡Total IgE	Tick bite history, Reaction to tick bite, Anaphylaxis
UNC0026	M	55	Control	0.02	34.9	Remote history of tick bites; consumes mammalian meat with no signs or symptoms associated
UNC0029	M	49	Control	0	78	Tick bite over 10 years prior; consumes mammalian meat with no signs or symptoms associated
UNC0039	F	50	Control	0	32	Denies a history of tick bites; consumes mammalian meat with no signs or symptoms associated
UNC0095	F	38	Control	0	<2	Denies a history of tick bites; consumes mammalian meat with no signs or symptoms associated
UNC0142	F	53	Control	0	12.8	Denies a history of tick bites; consumes mammalian meat with no signs or symptoms associated
UNC0216	F	28	Control	0.02	778	Remote history of single tick bite; consumes mammalian meat with no signs or symptoms associated
UNC0229	F	19	Control	0	2	Denies a history of tick bites; consumes mammalian meat with no signs or symptoms associated
UNC0233	F	31	Control	0.01	83.8	Tick bite over 15 years prior; consumes mammalian meat with no signs or symptoms associated
UNC0239	M	25	Control	0	19.9	Denies a history of tick bites; consumes mammalian meat daily with no signs or symptoms
UNC0240	M	54	Control	0	84.4	Remote history of tick bites; consumes mammalian meat regularly with no signs or symptoms associated
UNC0209	M	71	AGS	68	767	Endorses tick bite within past 1 yr; Large local skin reaction to tick bite; Anaphylaxis (GI, AE)
UNC0210	F	52	AGS	7.98	668	Endorses tick bite within past 1 yr; Large local skin reaction to tick bite; Anaphylaxis (U, AE, GI)
UNC0211	F	58	AGS	1.11	114	Endorses tick bite within past 1 yr; Large local skin reaction to tick bite; Urticaria
UNC0212	F	68	AGS	2.19	59	Endorses tick bite within past 1 yr; Large local skin reaction to tick bite; Anaphylaxis (U, GI, CV)
UNC0217	F	32	AGS	57.4	239	Endorses tick bite within past 6 mos; Large local skin reaction to tick bite; reports larval tick bites; Urticaria
UNC0218	M	29	AGS	15.7	152	Endorses tick bite within past 1 yr; Large local skin reaction to tick bite; Anaphylaxis (U, GI)
UNC0219	M	47	AGS	25.9	185	Endorses tick bite within past 1 yr; Large local skin reaction to tick bite; Urticaria
UNC0221	F	20	AGS	5.06	179	Endorses tick bite within past 1 yr; Large local skin reaction to tick bite; Anaphylaxis (U, GI, AE)
UNC0222	F	41	AGS	9.56	68.2	Endorses tick bite within past 1 yr; Large local skin reaction to tick bite; Urticaria
UNC0224	M	19	AGS	31.7	263	Endorses tick bite within past 2 mos; >100 larval tick bites; persistent itching at tick bite sites; Urticaria
UNC0225	F	26	AGS	0.13	129	Endorses tick bite within past 1 yr; Large local skin reaction to tick bite; Urticaria
UNC0230	M	33	AGS	14	497	Endorses tick bite within past 1 yr; Large local skin reaction to tick bite; Anaphylaxis (U, GI, CV)
UNC0231	M	45	AGS	0.59	121	Endorses tick bite within past 1 yr; Large local skin reaction to tick bite; Anaphylaxis (U, GI)
UNC0232	M	37	AGS	1.35	1464	Endorses tick bite within past 1 yr; Large local skin reaction to tick bite; Urticaria
UNC0234	F	46	AGS	0.75	102	Endorses tick bite within past 1 yr; Large local skin reaction to tick bite; Angioedema
UNC0235	F	27	AGS	6.47	201	Endorses tick bite within past 6 mos; Large local skin reaction to tick bite; Anaphylaxis (U, GI, CV)
UNC0236	F	31	AGS	2.87	159	Endorses tick bite within past 1 yr; Large local skin reaction to tick bite; Urticaria
UNC0237	M	49	AGS	13.5	227	Endorses tick bite within past 4 mos; Large local skin reaction to tick bite; Anaphylaxis (U, GI, AIR)

‡Quantified at the time single-cell RNA-sequencing; *sIgE – Alpha-gal specific IgE.

AIR – wheezing, throat tightness/closure, cough; AE – angioedema; CV – hypotension; GI – nausea, emesis, diarrhea, abdominal pain; U – urticaria.

**Figure 1:**
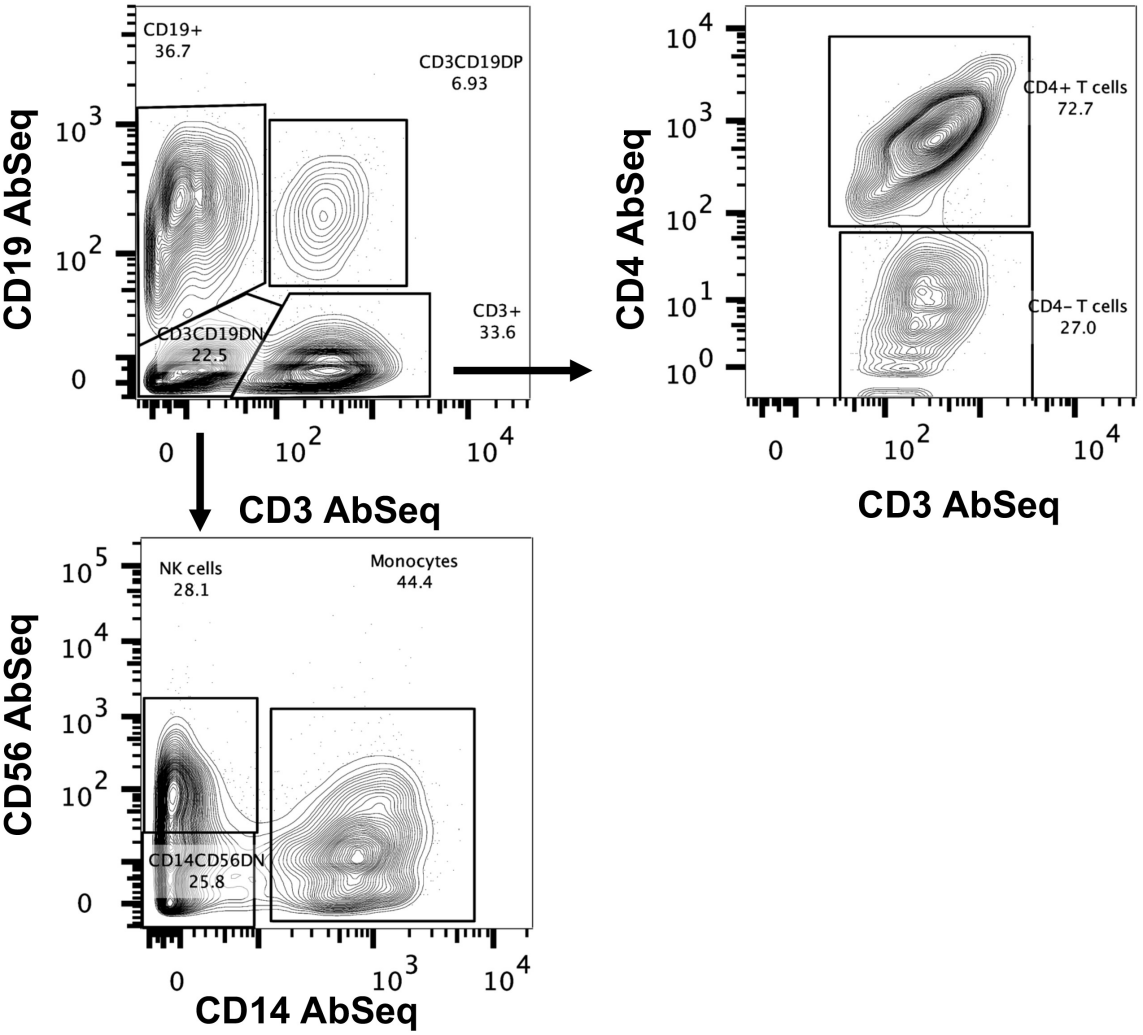
Identification of immune cells using BD AbSeq Ab-Oligos. Manual gating on the combined AbSeq dataset to identify major immune subsets, including T cells, B cells, NK cells and monocytes.

To detect rare B cell populations, including plasmablast and IgE-producing cells, the B cell population was enriched using negative selection to capture sufficient cell numbers. We routinely obtained 90% B cell enrichments (**Supplementary Figure S1**). PBMCs were spiked with enriched B cells at a 1:1 ratio before multiplexing and surface labeling with AbSeq Ab-O. We captured 437,770 cells for scRNA-seq, including 153,692 cells from controls (average 15,360 per subject, 37.4% of total cells) and 284,078 (average 15,782 per subject, 62.6% of total cells) from those with AGS. Cell population classification was performed using AbSeq Ab-O to identify T cells, B cells, NK cells, and monocytes (**Figure 1**). B cells (CD19**^+^** cells) constituted 36.7% of the total population, while T cells (CD3^+^ cells) constituted 33.6%. Among 22.5% of the CD3^-^CD19^-^ population, 44.4% were CD14^+^ monocytes, and 28.1% were CD56^+^ NK cells.

### An unsupervised weighted nearest-neighbor analysis integrating transcriptomes and surface proteins identified forty-three unique clusters

**Figure 2:**
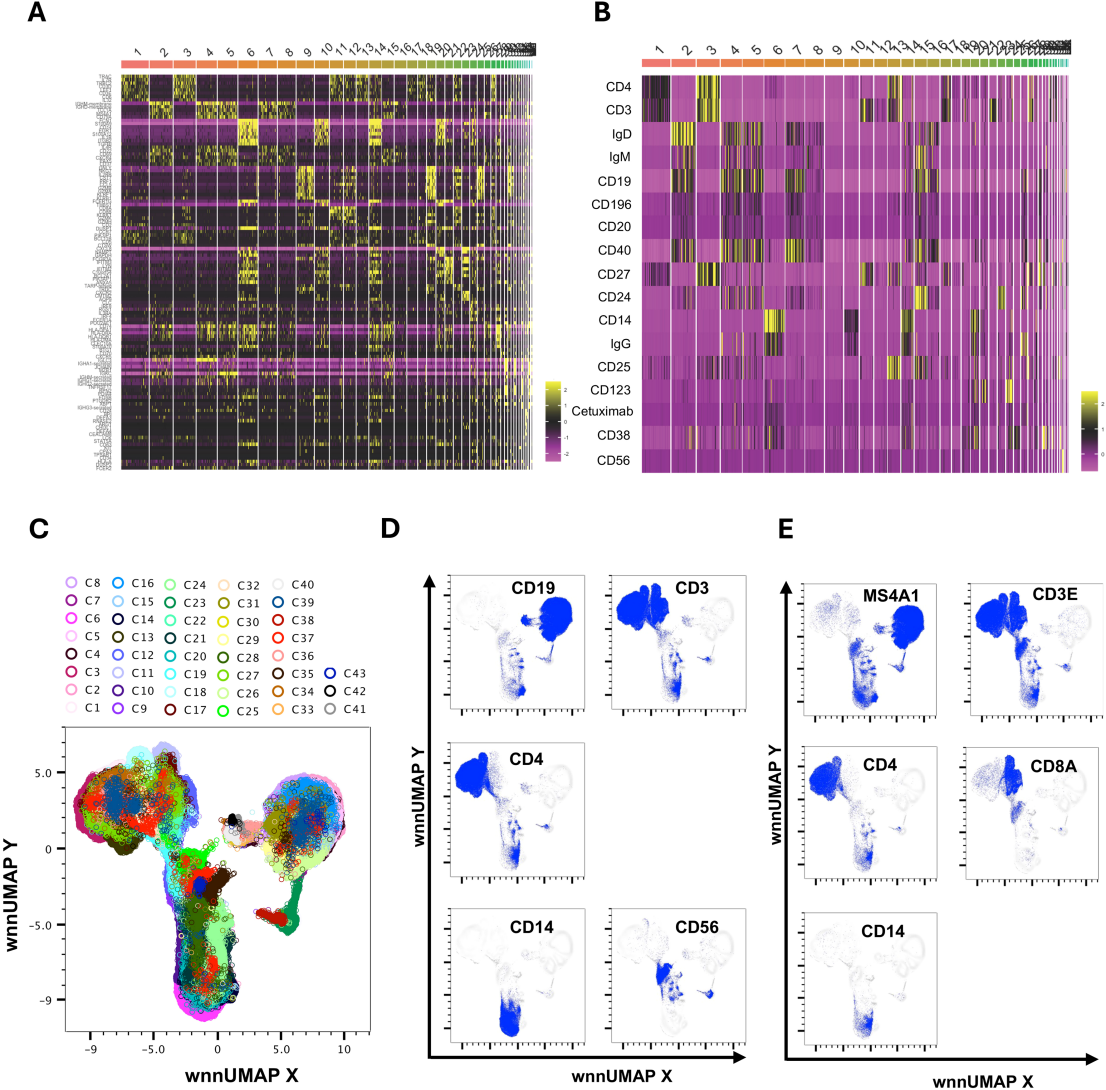
Multimodal markers to identify immune cell clusters. RNA heatmap **(A)** and antibody tags-derived (AbSeq Ab-Oligos) heatmap **(B)** of markers and their integration in WNN analysis led to the identification of 43 major WNN clusters **(C)**. Manual gating on AbSeq Ab-Oligos and mapping to WNN-UMAP based on AbSeq Ab-Oligos **(D)** and their corresponding gene transcript expression **(E)** to identify T cells, B cells, NK cells and monocytes.

**Figure 3:**
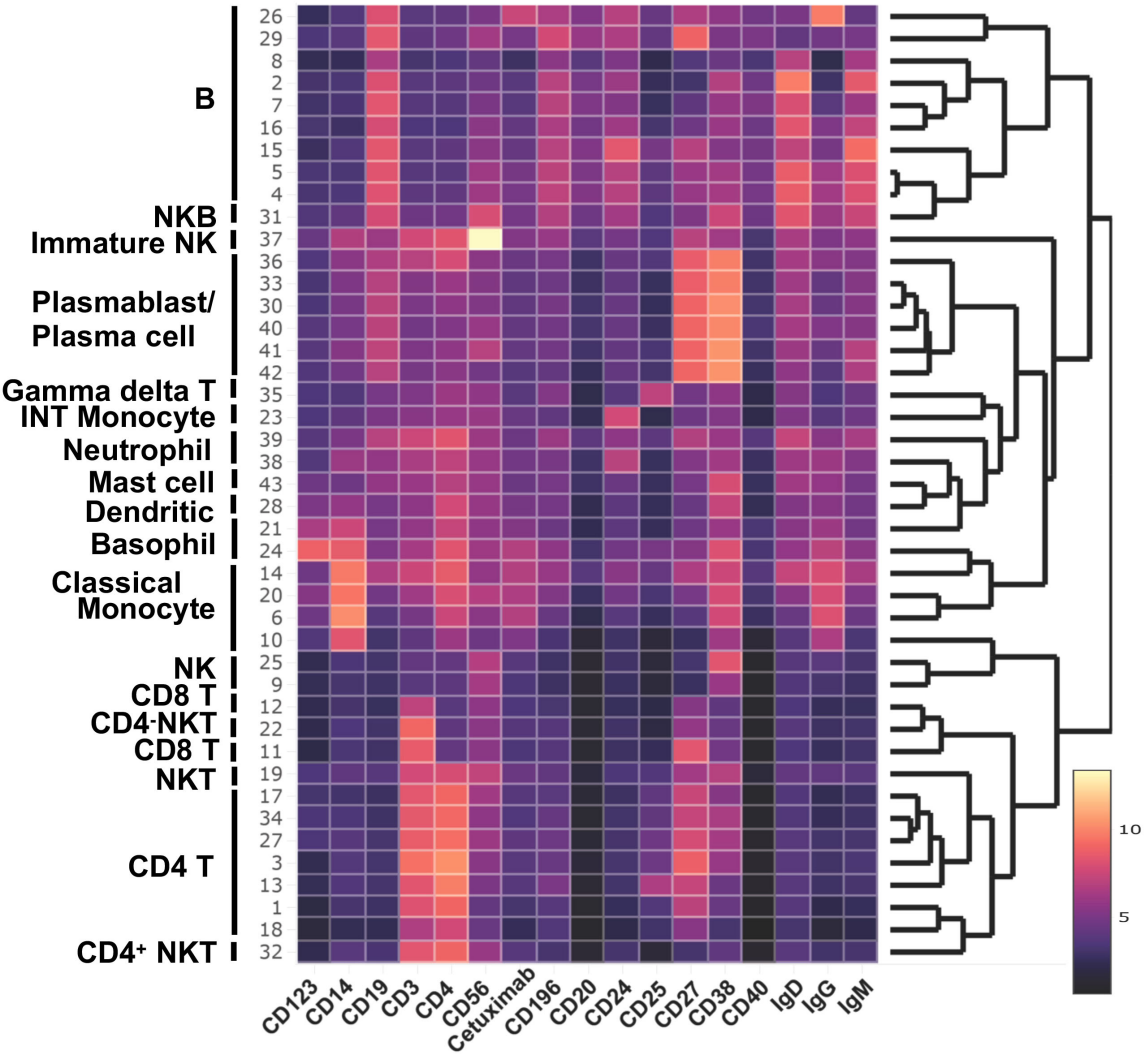
Major cell population identified in WNN clusters. Heatmap of protein makers (AbSeq Ab-Oligos) in WNN clusters.

We utilized the Seurat plug-in in SeqGeq for multimodal analysis of combined cells from both control and AGS subjects. The Seurat WNN analysis returned 83 distinct clusters based on PBMC transcriptomes and antibody tags (ADTs) associated with AbSeq Ab-Oligos. The plug-in output data included Uniform Manifold Approximation and Projections (UMAP) based on RNA, ADT, or WNN (**Supplementary Figure S2**), as well as RNA heatmap and antibody tags-derived heatmap (**Figures 2A, B**). Of the clusters identified, thirty-nine of them contained fewer than ten cells and were unable to be characterized either using protein markers or transcriptomes, so they were excluded from this analysis. The remaining 43 clusters comprised cells ranging from 29,501 to 178 and were subjected to more detailed analysis (**Figure 2C**). Interestingly, computation of visualization and mapping to WNN-UMAP based on either an established surface marker (AbSeq) or gene transcript marker resulted in a similar map for broad categories of immune cells, including B cells, T cells, NK cells, and monocytes (**Figures 2D, E**). Based on the surface antibody-derived signals (**Figure 3**) and transcriptomes (**Supplementary Figure S3**), we have identified all 43 clusters and have described them in greater detail. The percentage of cells from AGS subjects and controls in each cluster was also determined (**Supplementary Table S3**). We observed variability in clusters among AGS and control subjects (**Supplementary Figures S4**, **S5**). Clusters containing a lower number of cells generally have more variability than clusters with a higher number of cells.

### CD4^+^ T cells

**Figure 4:**
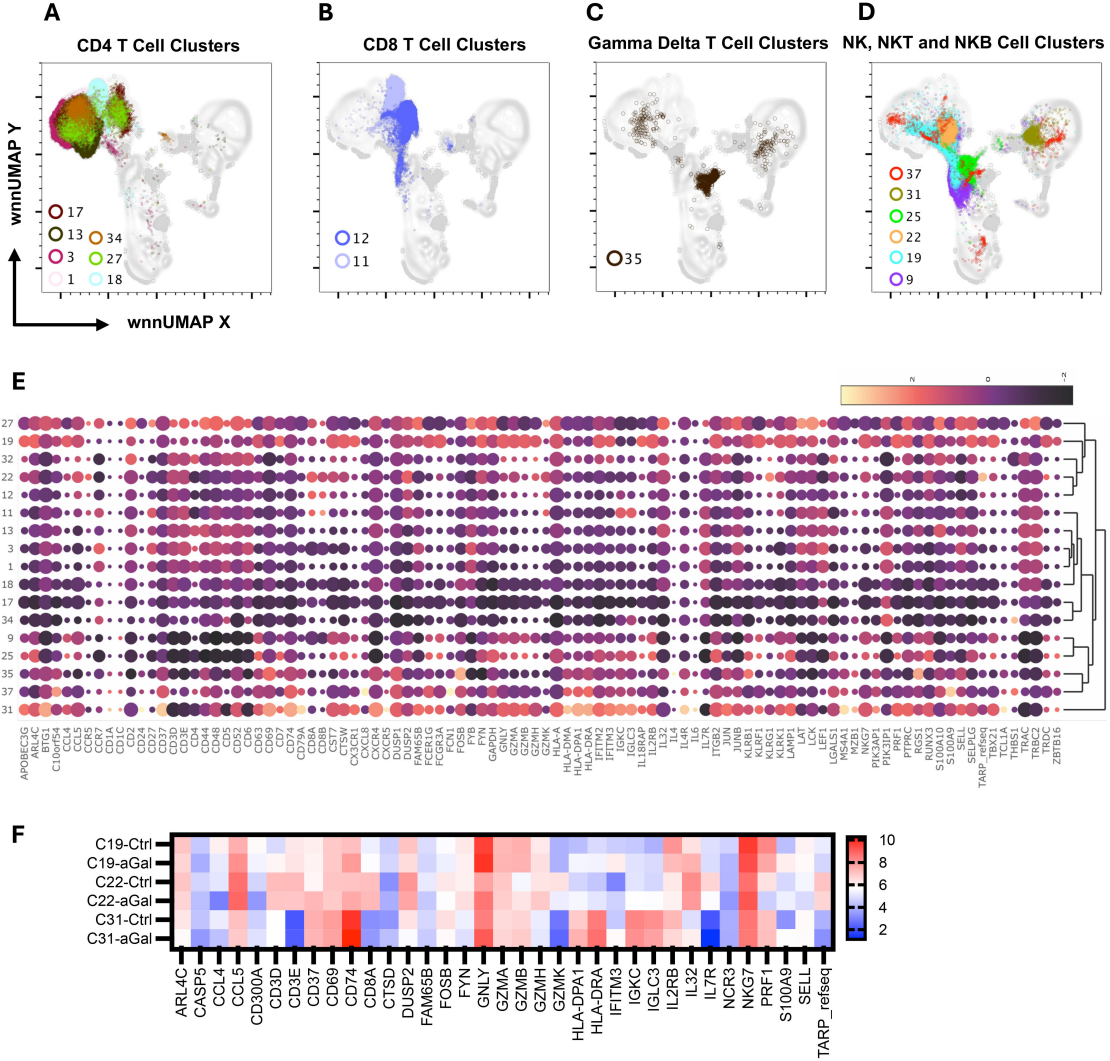
Multiomic analysis of the T cell and NK cell populations. WNN clusters that were identified as CD4^+^ T cell **(A)**, CD8^+^ T cell **(B)**, Gamma-delta T cell **(C)**, and NK, NKT, and NKB cells **(D)** based on protein and RNA markers. A heatmap depicting the differential expression of genes in the WNN cluster **(E)**. A heatmap showing differential gene expression in AGS subjects in comparison to controls **(F)**.

Clusters C01, C03, C13, C17, C18, and C34 were identified as CD4^+^ T cells (**Figure 4A**) based on the surface expression of CD3 and CD4 (**Figure 3**) as well as various amounts of transcription of *CD2, CD24, CD5, CD6, IL7R (CD127) LEF1, TRAC*, and *TRBC2*, (**Figure 4E**). C27 expressed surface CD4, but also had an elevated transcript of *IL32, CD8A*, and *CD8B*, and likely represents CD4, CD8 double-positive (DP) cells. The distribution of cells in each cluster was normal (less than 10% deviation from the expected cell number based on total cell number from alpha-gal and control subjects) except cluster C18, which was predominantly contributed by control subjects (81.5%), and C34, which was largely contributed by AGS subjects (90.3%) (**Supplementary Table S3**). C34 had high expression of *THBS1*, the ligand of CD36, which was upregulated in AGS subjects (**Table 2**). C13 contained cells with a higher expression of surface CD25 and 2.2-fold higher transcripts of *IL7R* (CD117) and, therefore, could be a regulatory CD4^+^ T cell cluster (**Figure 3**). The genes that were differentially upregulated (higher than 2-fold) in AGS participants included: *S100A9* in C13; *RUNX3* and *CD5* in C17, C34; *CD74* in C18; *CST7* in C18, *CD6* in C34 (**Table 2**). Upregulation was also noted for several genes, including *CCL5, CD247, RUNX3*, and *S100A9*, across T cell clusters in those with AGS when the cut-off was reduced to a 1.5-fold change.

**Table 2:** Upregulated genes in subjects with alpha-gal syndrome compared to controls.

Genes	Clusters with > 1.5-fold induction
***AQP9***	**Monocyte:** C20, C23
***BCL2A1***	**Monocyte:** C23
***CCL5***	**CD4 T:** C01, C03, C13, C17, C18, C32, C34; **NKT:** C19
***CD14***	**Monocyte:** C20
***CD37***	**Monocyte:** C20; **NKT:** C22
***CD5***	**CD4 T:** C01, C03, C13, C17, C18, C34
***CD52***	**Monocyte:** C20; **Basophil:** C21, C24; **Neutrophil:** C38; **Dendritic:** C28
***CD6***	**CD4 T:** C34
***CD69***	**B:** C29; **PB:** C30, C33
***CD74***	**B:** C8, C16; **CD4 T:** C1, C18, C27; **Gamma delta:** C35; **Neutrophil:** C38; **NK:** C9; **NKB:** C31; **NKT:** C19, C22, C32; **Monocyte:** C20
***CD79A***	**B:** C16, C29
***CD79B***	**B:** C8, C15, C26
***CD247***	**CD4 T:** C01, C03, C13, C17, C18, C34
***CD86***	**Monocyte:** C20
***CHI3L1***	**Neutrophil:** C38
***CTSD***	**Monocyte:** C10, C20; **Basophil:** C21; **Neutrophil:** C38
***CST7***	**CD4 T:** C18
***CXCL8***	**Dendritic cell:** C28; **Monocyte:** C10, C23; **Neutrophil:** C38; **NK (Immature):** C37
***CXCL16***	**Monocyte:** C6, C10, C20, C23; **Basophil:** C21
***DUSP1***	**Neutrophil:** C38
***FCER2***	**B:** C8, C16
***GNLY***	**Neutrophil:** C39; **NK:** C09; **NK (Immature)** C37; **NKB:** C31; **NKT:** C32
***HLA***	**CD4 T:** C03; **CD8 T:** C11; **PC/PB:** C30, C31, C32; **NK (Immature):** C37
***HLA-DMA***	**B:** C16; **Monocyte:** C20
***HLA-DPA1***	**B:** C16, C26; **Monocyte:** C20; **NKB:** C31; **NKT:** C19, C22
***HLA-DQB1***	**B:** C16, C26; **Monocyte:** C20
***HLA-DRA***	**B:** C16; **Monocyte:** C20; **Neutrophil:** C38; NKT: C22
***ICAM1***	**Monocyte:** C10, C20, C23
***IER3***	**Monocyte:** C10, C23
***IFITM3***	**Basophil:** C21, C24; **CD4 T:** C01, C03, C13, C18, C27, C32; **CD8T:** C11; C12, **Dendritic:** C28; **Monocyte:** C06, C10, C14, C20, C23; **NK:** C09, C25, C37; **NKB:** C31; **NKT:** C19, C22
***IL1B***	**Basophil:** C24; **Monocyte:** C20, C23
***IL32***	**NK (Immature):** C37; **NKB:** C31; **NKT:** C19; PC/PB C36
***JUNB***	**B:** C07, C16, C29; **CD4 T:** C1, C17, C34
***LAP3***	**Monocyte:** C10, 20
***LYN***	**B:** C07, C16, C29; **Monocyte:** C20; **Gamma delta:** C35
***NAMPT***	**Monocyte:** C23
***PTPRC***	**B:** C16
***RGS1***	**PC/PB:** C30, C33, C36, C40, C41, C42
***RPN2***	**Monocyte:** C6, C14; **PC/PB:** C30, C33, C36, C40, C41, C42
***RUNX3***	**Basophil:** C21, C24; **CD4 T:** C01, C03, C13, C17, C18, C34; **CD8T:** C11, C12; **Monocyte:** C10, C20
***S100A9***	**B:** C02, C04, C05, C07, C08, C15, C26; **CD4 T:** C01, C03, C13, C18, C27; **CD8 T:** C11, C12; **Gamma delta:** C35; **NK:** C09, C25, C37; **NKB:** C31; **NKT:** C19, C22, C32; **PC/PB:** C30, C33, C36, **Neutrophil:** C38
***THBS1***	**Basophil:** C24; **CD4 T:** C34; **Dendritic:** C28; **Monocyte:** C10, C20; **NK (Immature):** C37
***TLR2***	**Monocyte:** C20
***TREM1***	**Monocyte:** C23

Bold = immune cell of cluster origin.

### CD8^+^ T cells

Since our panel for surface staining did not include CD8 AbSeq Ab-O, we identified CD8 T cell clusters based on positive surface CD3 expression, negative CD4 expression, and the presence of *CD8A* transcriptomes (**Figures 3**, **4B**). We identified C11 and C12 as CD8 T cell clusters (**Figure 4B**) that contained transcripts of *CCL5, CD8A, GZMH, GZMK, IL7R, KLRK1, TRAC* and *TRBC2*, at various levels (**Figure 4E**). In AGS subjects, limited differential gene expression in CD8 T cells were observed and included *HLA*, *RUNX3*, and *S100A9* (**Table 2**).

### Gamma delta (γδ) T cells

C35 had an upregulated *TRDC* along with *KLRB1*, indicating it is gamma delta (**γ**δ) T cells (**Figures 4C, E**). This cluster had a very high surface expression of CD25 (9.5X) along with transcripts of *FOSB* and *RGS1* (**Figures 3**, **4E**). Upregulation of *CD74* and *S100A9* was observed in AGS in comparison to the control (**Table 2**).

### Natural killer cells, NK T cells, and NK B cells

C9 and C25 were two NK cell clusters containing the CD56^dim^ surface expression and higher *FCGR3A* transcripts, depicting a typical peripheral blood NK cell population (**Figures 3**, **4D, E**). The clusters contained various levels of transcripts of *CCL5, CST7, GNLY, GZMA, GZMB, IL2RB, KLRB, KLRF1, NKG7*, and *PRF1*, associated with NK cell cytotoxicity. No differential gene expression was observed in AGS subjects except for the upregulation of *S100A9* and *IFITM3* (**Table 2**).

C19 was recognized as an NKT cell cluster, characterized by surface expression of CD3 and CD56 and upregulated transcripts of *FCGR3A* and *NKG7* (**Figures 3**, **4D, E**). The cluster also contained *TRDC* that participates in antigen recognition, including lipids presented by MHC-like protein CD1D and phosphoantigens presented by butyrophilin-like molecules (30). The cluster has low levels of CD4 transcripts. Consistent with the role of CD4^-^NKT cells in Th1 response, transcription factor *TBX21* was upregulated (31). Several cytotoxic effector molecules, including *GNLY* (10.6X), *PRF1* (5.7X), *GZMA, GZMB*, and *GZMH* were also upregulated. The cluster contained both inhibitory receptors *KLRB1* and *KLRG1* and activating receptors *KLRF1* and *KLRK1*, although the activating receptors outweighed the inhibitory receptors. Other upregulated genes included those involved in leucocyte adhesion and trafficking (*CCL4*, *CCL5, CX3CR1, ITGB2, SELPLG)*, inflammation (*IL18RAP, IL32*), and immunomodulation (*CTSW, CST7, IFITM2, PTPRC*). C19 contained a high percentage of cells from AGS subjects (77.9%), and upregulation was further noted for several genes, including *CCL5, CD74, HLA-DPA1, IFITM3, IL32*, and *S100A9* (**Table 2**, **Figure 4F**). The genes that were downregulated included *CCL4, DUSP2, KLRF1* and *PRF1*.

C22 was another CD4^-^NKT cell cluster; however, it contained the TCR gamma alternate reading frame transcript (*TARP_refseq)* (**Figure 4E**). This cluster showed the highest number of genes that were either upregulated (116 genes) or downregulated (66 genes) significantly in AGS subjects (data not shown). The genes that were downregulated more than two-fold included *ARL4C, CD3D, CD3E, CD8A, CD8B, CST7* (4.8X), *CTSW* (4X), *CCL5* (6.2X), *DUSP2* (4.1X), *GZMA* (4.7X), *GZMH* (5.5X), *IL2RB* (2.5X), *IL32* (3X), *KLRB1*(3.5X), *KLRK1*(4.2X), *LCK, NKG7* (3.9X), *PRF1*(3.3X), *TARP_refseq* (17X); many of those genes are involved in cell-mediated killing (**Figure 4F** and data not shown). In contrast, the genes that were upregulated were mostly involved in antigen presentation and included *CD37* (2.5X), *CD74* (6.3X), *HLA-DPA1* (4.7X), *HLA-DRA* (15X), *IGKC* (8.6X), *IGLC3* (8X) and *S100A9* (10.7X).

C37 had a very high surface expression of CD56 (208X, **Figure 3**), a feature of immature NK cells (32). C37 was an unusual cluster, containing 24% CD19^+^ cells, 47% CD4^+^ cells, and 28% CD3^-^CD19^-^ cells, based on the surface expression of CD3 and CD19 (**Figure 4D** and data not shown). The cluster had lower transcripts of *GZMA, GZMB, GZMH, GZMK*, and *PRF1*, while higher transcripts of *CD37, CXCL8, FCN1, HLA-DMA, HLA-DPA1*, and *HLA-DRA* were consistent with immune regulatory function. It is interesting to note that *FCN1* is an extracellular lectin that functions as a pattern-recognition receptor and binds to the sugar moiety of pathogen-associated molecular patterns (PAMPs), activating cells and inducing chemokines, including CXCL8 (33). We noted the induction of *CXCL8* along with *GNLY, IL32* and *THBS1* in this cluster in AGS subjects.

C31 contained cells that had features unique to both NK and B cells and were defined as NKB cells (**Figures 3**, **4E**). NKB cells expressed B cell receptors, including CD19, IgD, IgM, CD24, CD38, and CD196, on their cell surface, along with the NK marker CD56. The NKB cells carried various transcriptomes necessary for efficient B cell function such as (i) *CD1A* and *CD1C* necessary for the recognition of glycolipid antigen, (ii) major histocompatibility complex (MHC) class II molecules *HLA-DMA, HLADPA1*, and *HLA-DRA* important for antigen presentation and (iii) *CD79A, CD79B, FCER2, IL4R, IL-6*, *and MZB1* for efficient for BCR signaling and differentiation of B cell into immunoglobulin-secreting cell. Various transcripts unique to NK cell and their cytolytic function, such as *FCGR3A*, GZMA, *KLRB1, KLRK1, KLRF1, NKG7*, and *PRF1*, were also upregulated. Most cells (84.4%) in this cluster were contributed by AGS subjects. Interestingly, upregulation of *CD74, HLA-DPA1, IFITM3, IL32*, and *S100A9* was further noted in AGS subjects like C19 (**Figure 4F**). The genes that were downregulated were *DUSP2, CCL4, FOSB*, IgHG1 and *IL2RB*. The downregulation of *IGHG1_secreted* and upregulation of *GNLY* and *IL32* suggest it could have cytotoxic activity against the pathogens present in tick saliva.

### Monocytes

**Figure 5:**
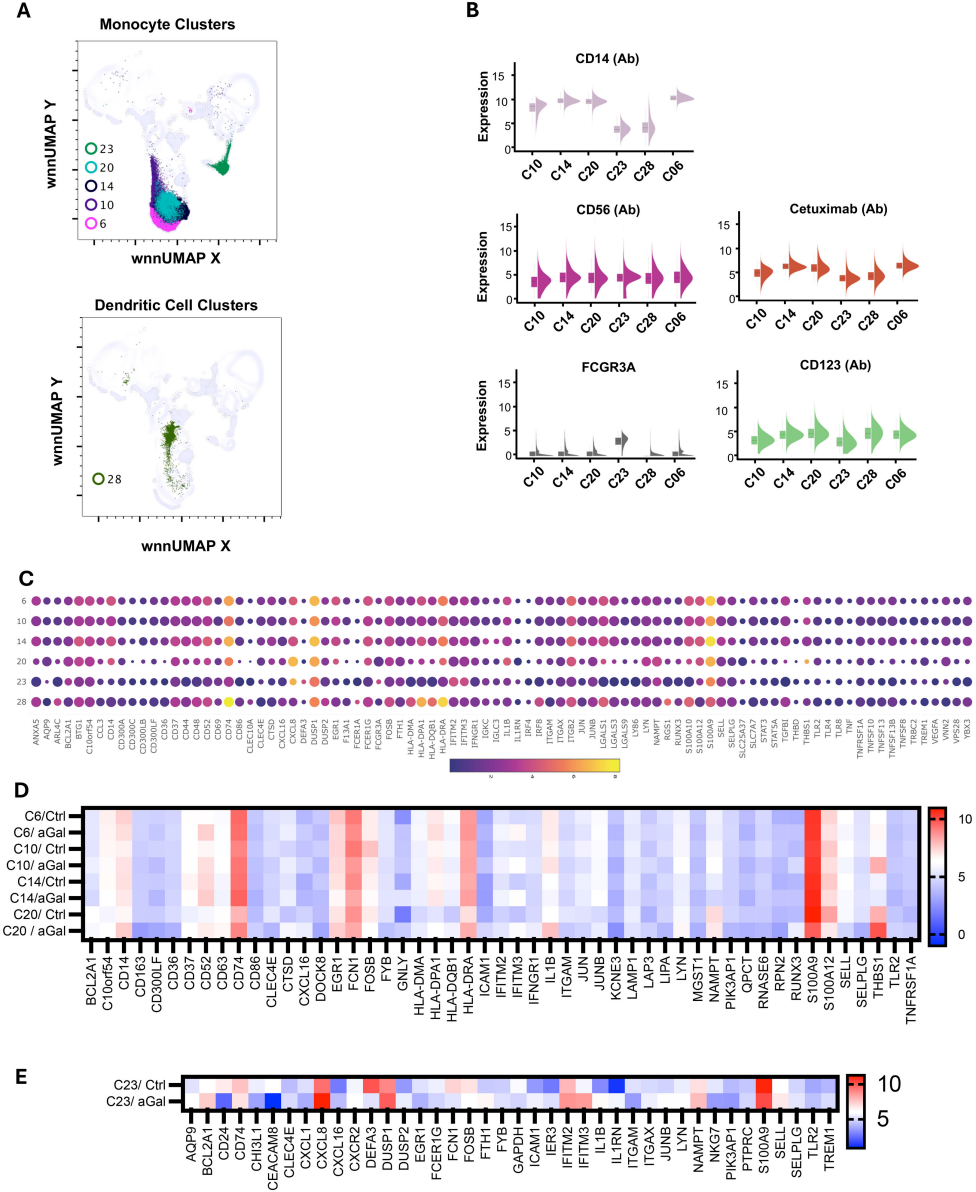
Multiomic analysis of monocyte and dendritic cell populations. WNN clusters that were identified as monocytes and dendritic cells **(A)**. Violin plots show the differential expression of surface markers **(B)**. Bubble heatmap showing differential gene expression in monocyte and dendritic cell clusters **(C)**. Heatmaps showing differential gene expression in AGS subjects compared to controls **(D, E)**.

C06, C10, C14, C20, and C23 were identified as monocytes (**Figure 5A**). We used *FCGR3A* (CD16a) transcripts along with the surface expression of CD14 and CD56 to identify different subsets of monocytes (**Figure 5B**). We identified four clusters of cells with high CD14 and low *FCGR3A* expression, which is characteristic of classical monocytes (CM). CM clusters (C06, C10, C14, and C20) exhibited distinct gene expression patterns, particularly showing elevated levels of *CCL3, CD14, CD36, CXCL8, DUSP1, EGR1, FCN1, FCER1G, IL1B, S100A9, S100A12* and *THBS1*, although the expression levels varied across the clusters (**Figure 5C**). Additionally, we observed increased expression of *NAMPT, BCLA2, TGFBI, TLR2*, and members of the TNF family, such as *TNFRSF1A, TNSF10, TNFSF13*, and *TNFSF13B*. The elevated level of lipopolysaccharide (LPS) receptor *TLR2*, LPS coreceptor *CD14*, LPS sensor *CD36*, alarmins *S100A9*, and inflammatory cytokines *IL1B* are consistent with the innate response of monocytes to damage-associated molecular patterns (DAMPs) and PAMPs (34–36). The presence of *DUSP1*, a negative regulator of inflammatory cytokines, *BCLA2*, which encodes an anti-apoptotic protein, and *TNSF13* and *TNSF13B*, which are involved in stimulating B- and T-cells and regulating humoral immunity, suggests that these cells have the capability to modulate the transition from innate to adaptive immunity (37–39). In AGS subjects, several genes including *CD52, CXCL16, HLA-DPA1, HLA-DRA, ICAM1, IFITM3, LAP3*, and *THBS1* were upregulated, while *CD300LF, CLC4E, S100A12, RNASE6*, and *RPN2* were downregulated in several CM clusters (**Figure 5D**).

There was a slight increase in the *FCGR3A* transcript in cluster C20, along with increased expression of genes encoding pro-inflammatory mediators such as *CXCL8* (20-fold), *IL1B* (9-fold), and *THBS1* (36-fold). This suggests that the cluster was transitioning to intermediate monocytes (INT). INT monocytes have a higher capacity to secrete inflammatory cytokines, and an increase in their proportion within monocyte subsets is associated with an inflammatory environment (40). It was observed that 82% of cells in C20 came from individuals with AGS. Additionally, most of the differential gene expression also occurred in this cluster, including upregulation of *CD14, CD37, CD52, CD74, CD86, CTSD, EGR1, HLA-DPA1, HLA-DQB1, HLA-DRA, IL1B, THBS1*, and *TLR2* in AGS subjects (**Figure 5D**). The genes that were downregulated intensively in this cluster in AGS subjects included *CD36, FOSB, S100A9, SELL*, and *SELPLG* (**Figure 5D** and data not shown). The increased expression of *CD74*, along with MHC class II molecules, clearly demonstrates their specialization in antigen presentation and transition to M2 macrophages in AGS subjects.

C23 contained monocytes with CD14^dim^CD56^-^ surface phenotype and carried transcripts of *FCGR3A*, suggesting that they were nonclassical monocytes (NCM) or patrolling monocytes (**Figure 5B**). In contrast to CM/INT clusters, the upregulation of *IL1RN* occurred in this NCM cluster, which is associated with the inhibition of *IL1B*, consistent with the anti-inflammatory response of NCM (41, 42). In C23, most of the cells (97%) were contributed by AGS, and further upregulation of *BCL2A1*, CXCL8, and *NAMPT* was observed (**Figure 5E**).

### Dendritic cells

The C28 cells showed high levels of *CLEC10A, HLA-DPA1, HLA-DRA, HLA-DQB1, HLA-DMA*, and *CD74* genes, which are characteristic of antigen-presenting dendritic cells (**Figures 5A, C**). Additionally, we noticed increased expression of *ARL4C, CXCL16, FCER1A, FOSB, IRF8, FLT3, LGALS1, RGS1* and *S100A1* transcripts in this cell cluster. C28 demonstrated normal cell representation in control and AGS subjects, with upregulation of *CD52* and *IFITM3* noted in the AGS subjects (**Table 2**).

### Basophils

**Figure 6:**
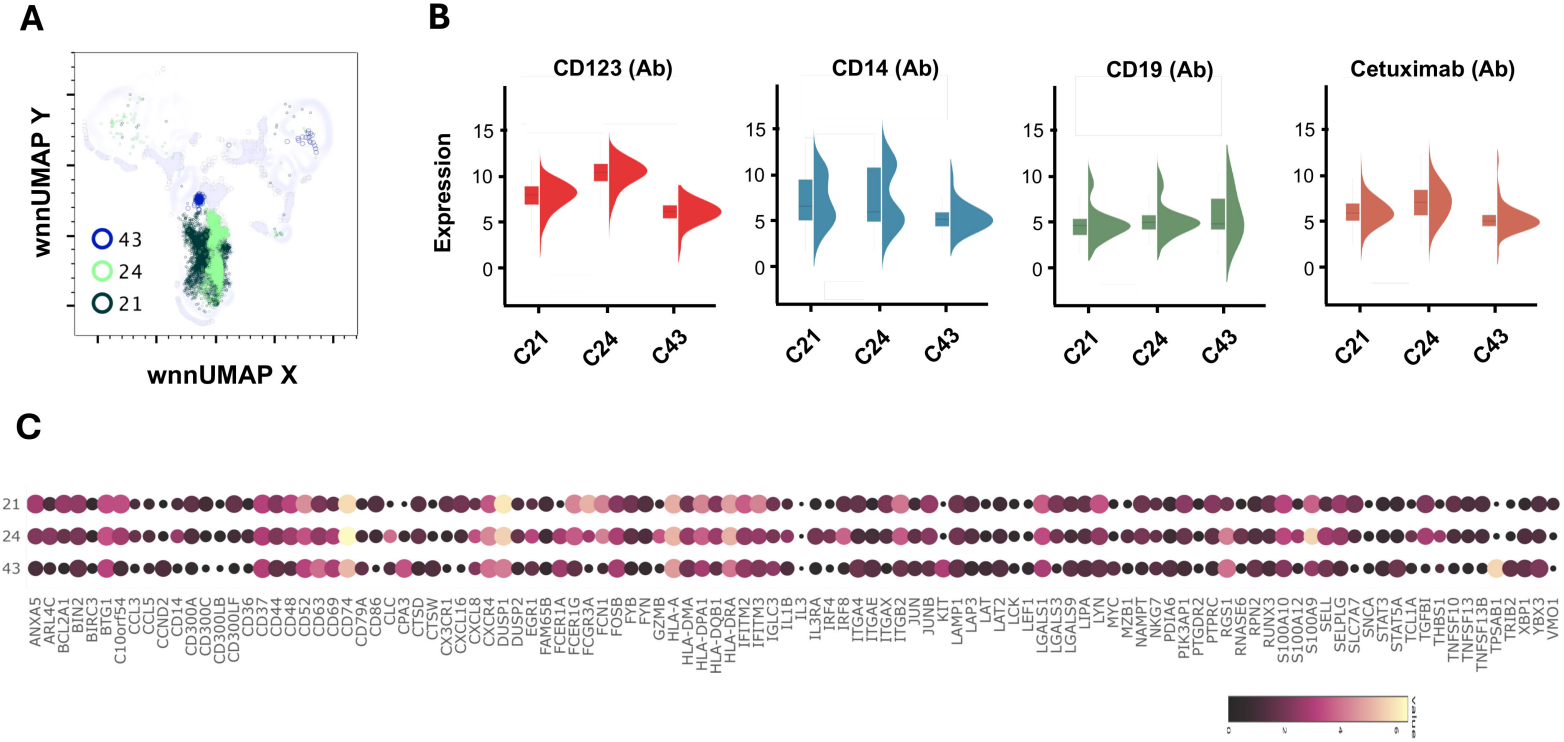
Multiomic analysis of basophil and mast cell populations. WNN clusters C21 and C23 were identified as basophils and C43 as the progenitor mast cell population **(A)**. Violin plots showing the differential expression of surface markers **(B)**. Bubble heatmap showing differential gene expression **(C)**.

C21 and C24 expressed 4.9-fold and 40-fold higher surface expression CD123 and were recognized as the basophil clusters (**Figures 3**, **6A, B**). Interestingly, both clusters showed the presence of anti-alpha-gal specific antibodies on the surface (detection via oligo-labeled cetuximab, a chimeric monoclonal antibody containing alpha-gal on immunoglobulin F(ab) fragments). While C24 had a normal distribution of cells between AGS- and control subjects, C21 had a higher contribution of cells (73%) from AGS subjects. We observed a large variation in signature genes in these two clusters (**Figure 6C**). C24 had a higher transcript of *CD14, CD69, CD74, CLC, CXCL8, EGR1, IL1B, IRF4, IFR8, RGS1, S100A9*, and *THBS1*. In contrast, C21 had a higher *ANAX5, BCL2A1, CD52, CXCL16, DUSP1, FCER1G, FCGR3A, FCN1, IFITM3, ITGB2, LYN*, and *PTPRC.* In AGS subjects, upregulation of *CD52, CTSD, CXCL16* and *IFTIM3* was observed in C21 and *CD52, CXCL8, EGR1, IFITM3, IL1B, NKG7, RUNX3*, and *THBS1* in C24 (**Table 2**). Further in C24, a 1.8-fold decrease in FCER1A expression was observed in AGS subjects; however, no significant difference was found in FCER1G.

### Mast cells

C43 contained cells with markedly increased *KIT* (8-fold), *TPSAB1* (Tryptase, 53-fold), and *CPA3* (Carboxypeptidase A3, 10.81-fold), which are characteristic of mast cells (**Figures 6A, C**). We further observed high *FCER1A* (4.8-fold) and *CD63* (5.8-fold) in C43. The cluster had upregulated *CXCR4, FOSB, IGLC3, ITGAE, LGALS1, LGALS9*, *RGS1*, and *STAT5A*. *LGALS9* is an inhibitory member of the galectin family and specifically inhibits Th1 and Th17 cells while simultaneously promoting the differentiation of regulatory T cells (Treg) (43, 44). The cluster mainly consisted of cells (89%) from subjects with AGS and showed a four-fold increase in *IFITM3* compared to the control subject (**Table 2**). In AGS subjects, FCER1A expression decreased by 2.5 times; however, there was no significant difference in FCER1G.

### Neutrophils

**Figure 7:**
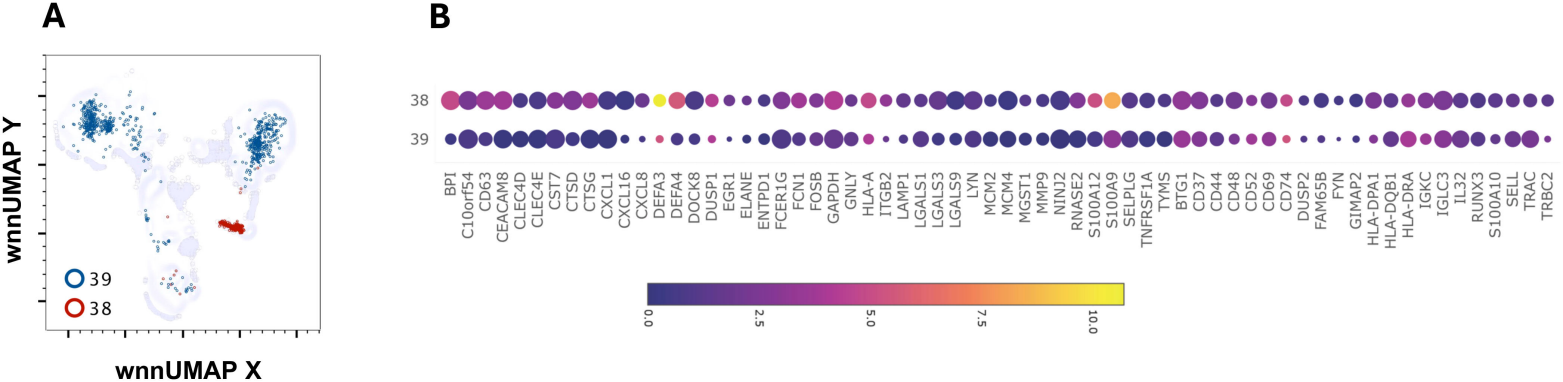
Multiomic analysis of neutrophil populations. WNN clusters that were identified as neutrophils **(A)**. Bubble heatmap showing differential gene expression **(B)**.

C38 had a characteristic signature of granulocytes- a very high *DEF3* (986-fold), *DEF4* (49-fold), *BPI* (29-fold) and *CEACAM8* (13-fold) (**Figures 7A, B**). It also contained higher*, ARG1, CHI3L1, CTSD, CD24, CD63, DUSP1, FCN1*, *NKG7* and *S100A9, S100A12* (**Figure 7B** and data not shown). Eighty-three percent of cells in this cluster were from AGS subjects. Several transcripts such as *CD52, CD74, CH3L1, CXCL8, DUSP1, HLA, HLA-DRA*, and *S100A9* were further upregulated in AGS. CXCL8 (IL-8) is a potent chemokine that attracts and activates neutrophils, regulates other leukocytes, and promotes wound healing (45–47). High concentrations of CXCL8, however, repel neutrophils away from the site of inflammation, indicating that it may have both pro-inflammatory and anti-inflammatory effects (48). C39 also contained eight-fold higher *DEF3*, suggesting it could be a granulocyte cluster. However, the cluster was not discrete and contained cells having surface expression of CD3, CD4 and CD19, as well as transcripts of *CD3D, MS4A1*, and *CD79A* (data not shown).

### Multi-omics analysis reveals distinct B cell subpopulations, including alpha-gal-specific memory cells

**Figure 8:**
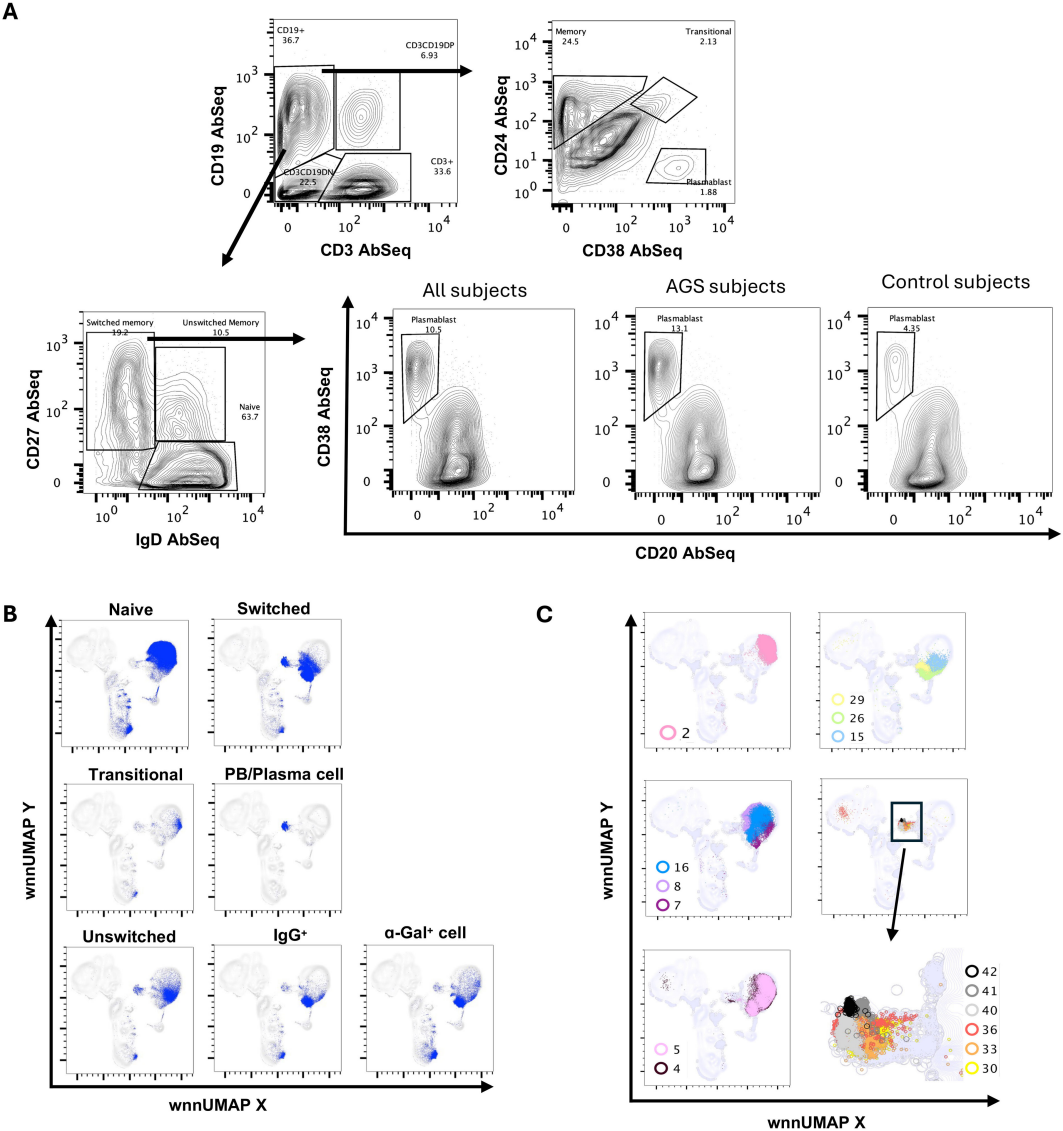
Multiomic analysis of B cells. Manual gating on AbSeq Ab-O to identify B cell subsets **(A)**. Manual gating on AbSeq Ab-O and mapping of B cell to WNN-UMAP **(B)**. WNN clusters that were identified as B cell populations **(C)**.

The enrichment of B cells by negative selection allowed the capture of more than 160,000 B cells for integrated WNN analysis. AbSeq surface staining employing canonical B cell surface markers CD19, IgD, IgM, IgG, CD20, CD24, CD25, CD27, and CD38 allowed discrimination of the different subpopulations of B cells including naïve (CD19^+^IgD^+^CD27^-^), transitional (CD19^+^CD24^++^CD38^+^), unswitched memory (CD19^+^CD27^+^IgD^+^) and switched memory (CD19^+^CD27^+^IgD^-^) (**Figure 8A**). Further, low-abundance plasmablast (PB)/plasma cell (PC) (CD19^+^CD27^+^IgD^-^CD20^-^CD38^++^) were also identified. Interestingly, a higher percentage of plasmablasts was observed in AGS in comparison to control subjects, despite similar naïve, unswitched memory and switched memory B cell subpopulations (**Figure 8A**) (data not shown). We mapped these subsets of B cells to WNN-UMAP (**Figure 8B**).

**Figure 9:**
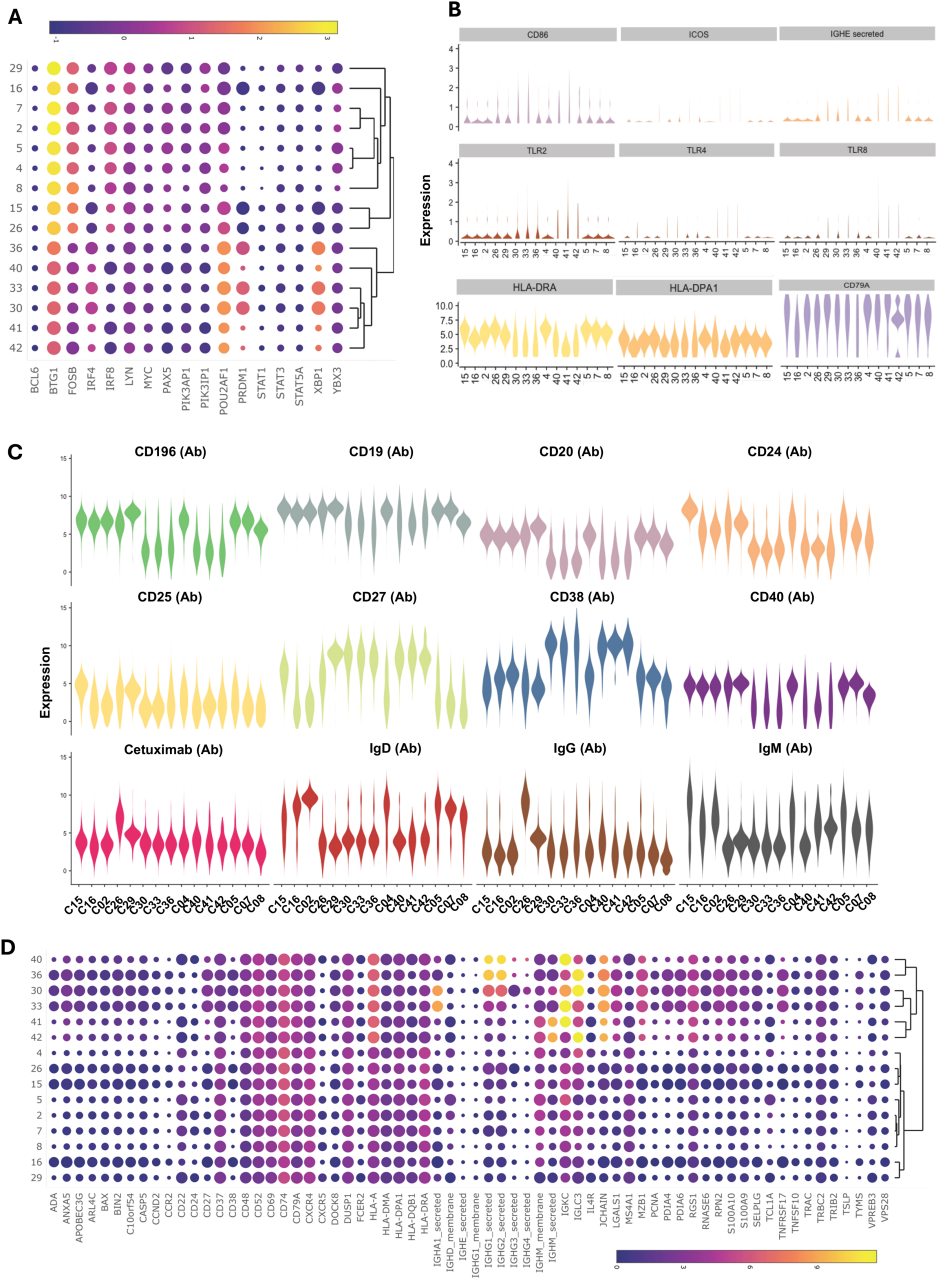
Multiomic analysis of B cells (continued). Bubble heatmap showing the differential gene expression of transcription factors **(A)**. Violin plots showing differential expression of genes in B cell clusters **(B)**. Violin plots showing the differential expression of surface protein markers **(C)**. Bubble heatmap showing differential gene expression **(D)**.

Multi-omics analysis using integrated WNN analysis identified 15 distinct clusters of B cells, namely, C2, C4, C5, C7, C8, C15, C16, C26, C29, C30, C33, C36, C40, C41, and C42. These clusters were also mapped to WNN-UMAP (**Figure 8C**) and compared to the classification of cells based on canonical surface markers. C2 constituted exclusively naïve B cell population (>97%). Clusters C7, C8, and C16 were largely populated by naïve B cells (>70%) but also contained memory cells (18%-24%). C4 and C5 had mixed populations of naïve (~62%), transitional (~3%), unswitched memory (~14%) and switched memory (~22%) cells. Clusters C4 and C5 had higher MHC class II transcripts, including *HLA-DR and HLA-DPA1*, as well as transcripts of costimulatory molecules *CD79A, IL4R*, and transcription factor *POU2AF1*, suggestive of an activated nature (**Figures 9A, B**). While C4 had higher *IGLC3*, C05 had higher *IGKC*. C15 had a higher expression of surface CD25 with an overall phenotype of IgD^+^IgM^+^CD24^high^CD25^high^CD27^+^CD19^+^, a characteristic of activated antigen-presenting B cells (**Figure 9C**). Interestingly, they also expressed higher transcripts of *CD79A, HLA-DR, CXCR4*, *IRF4, POU2AF1, PRDM1, and XBP1*, suggestive of their late activated B cell status and may be at the cusp of transitioning to PB (49). C16, which still had IgD^high^, IgM^+^ phenotype, however, showed upregulation of several genes in AGS subjects, including *CD79A, FECR2, HLA-DNA, HLA-DPA1, HLA-DQB1, HLA-DRA, JUNB* and *TLR4* (**Table 2**) and like C15 had high *IRF4, POU2AF1*, and *XBP1* suggestive of its activated status. C26 exclusively expressed surface IgG and alpha-Gal specific immunoglobulins (Ig) and was predominantly memory B cells (switched memory 86%, unswitched memory 6%). C26 contained high *HLA-DRA* and *POU2AF1* (**Figures 9A, B**), and upregulation of *CD79B*, MHC II molecules *HLA-DPA1 and HLA-DQB1*, and *S100A9* were observed in AGS subjects. C15, C16, and C26 contained elevated thymidylate synthetase (*TYMS*), indicative of their highly proliferating nature. C29 contained a switched memory B cell population (CD19^high^CD20^high^CD27^high^CCR6^high^ CD40^+^), with lower expression of IgG and alpha-gal expression. It also notably showed the presence of a low-level transcript of IGE_secreted. In addition, C29 exhibited a higher expression of *BTG* along with *CBLB*, suggesting a negative regulation of BCR and a transition of activated B cells or PB cells to memory B cells (**Figures 9A**, data not shown) (50, 51). Most cells in C29 were contributed by AGS subjects.

WNN analysis also identified six clusters of PB/PC, namely C30, C33, C36, C40, C41, and C42, which were all CD27^+^CD38^+^CD20^-^CD24^-^IgD^-^CCR6^low^ (**Figures 8C**, **9C**). These clusters exhibited lower levels of *PAX5* and *IRF8*, and higher levels of *POU2AF, IRF4, XBP1, PRDM1*, and *TNFRSF17.* C30, C33, and C36 showed significantly elevated levels of *PRDM1* and *XBP1*, along with *TYMS*. This is consistent with antibody-secreting and highly proliferative PB (49). Further, we observed upregulated *ADA, APOBE*C3G, *HLA, LGLS1, MZB1, PDIA4, PDIA6, RGS1, S100A9*, and *S100A10* and in all PB/PC clusters (**Figure 9D**). *CD196* (CCR6) surface expression was downregulated in all PB/PC clusters, including C30, C33, and C36. C30 and C33 were primarily IgA-secreting PB/PC, C36 and C40 were IgG1-, IgG2- and IgG3- secreting PB/PC and C41 and C42 were IgM-secreting PB/PC. IgG4-secreting transcripts were present in clusters C30, C33, C36, and C40. We did not find a PB/PC cluster that exclusively secretes only one class of immunoglobulin (Ig). Instead, we found IgE-secreted transcripts, albeit at a low level in C30, C40, C41 and C42 (**Figure 9B**). Interestingly, all these clusters had elevated transcripts for *TLR2, TLR4*, and *TLR8*, which could synergistically activate BCR and induce Ig class switch recombination (CSR). Most cells in clusters C30, C33, and C36 were contributed by a few AGS subjects (UNC222, UNC225) reporting recent tick bites.

### Single-cell analysis of IgE-secreting cells identified alpha-gal-secreting cells and revealed that multiple antibodies could be produced from a single B cell

**Figure 10:**
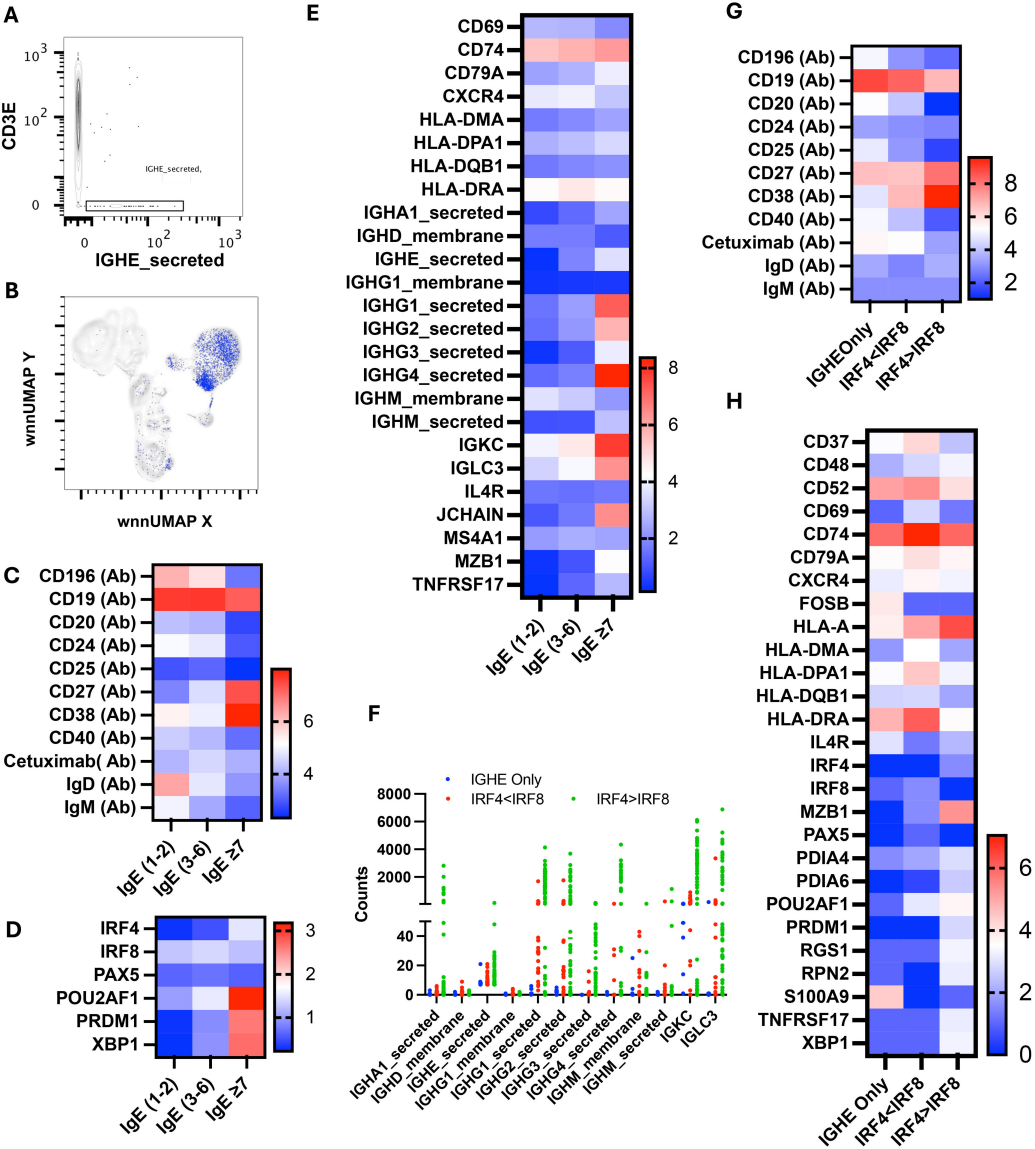
Multiomic analysis of IgE-secreting B cells. Manual gating on IgE-secreted RNA to identify IgE-secreting B cells **(A)** and mapping it to WNN-UMAP **(B)**. Heatmaps showing the surface protein markers in sub-sets of the IGHE-secreted population **(C)**. Heatmaps showing differential gene expression of B cell-specific transcription factors **(D)**, and antigen presentation and immunoglobulin secretion **(E)**. Immunoglobulin class transcriptomes count in the IGHE-secreted population **(F)**. Surface expression of B cell markers **(G)** and differential gene expression **(H)** in cells containing more than seven counts of IGHE-secreted transcripts.

WNN analysis discovered 1,141 cells containing a total of 11,017 IgE-secreted transcripts that we mapped to WNN-UMAP (**Figures 10A, B**). As expected, IgE-secreted transcript populated the B cell clusters. Based on the copies of IgE-secreted transcript per cell, we divided IgE-producing cells into three populations: (i) IgE_1–2_ having one to two copies, (ii) IgE_3–6_ having three to six copies, and (iii) IgE_≥7_ having seven or more copies. The IgE_≥7_ population resembled more like a PB/PC with a high surface CD27 and CD38 and a low CD20, CD24, and IgD (**Figure 10C**). Further transcription factors associated with B cell activation and immunoglobulin production, such as *POU2AF1, IRF4, PRDM1*, and *XBP1*, were upregulated, while transcription factors associated with resting naïve/memory cells, such as *PAX5* and *IRF8*, were downregulated (**Figure 10D**) (49). These cells also expressed lower surface CD196 and higher transcripts of *IGKC, IGLC3, MZB1*, and *TNFRSF17* (**Figures 10C–E**).

Interestingly, IgE_3–6_ population had the highest level of cell surface anti-alpha-gal specific Ig among these three sub-populations. These cells had the highest level of *HLA-DRA* as well as upregulated *CD74, IGKC*, and IGLCE, indicating a role in antigen presentation and BCR crosslinking (**Figure 10E**). Transcription factors *IRF4, POU2AF1, PRDM* (Blimp) and *XPB1* were expressed at an intermediate level and were higher than IgE_1-2_. We observed intermediate expression of IgD, IgM, CD27, and CD38 in the IgE_3–6_ population, suggesting that cells had not yet transitioned to PB/PC.

We further analyzed the IgE_≥7_ population and found that only eight out of 85 cells predominantly contained *IGHE-secreted* transcripts. All other cells have multiple transcripts of several Ig classes, including *IGHM_secreted*, *IGHA1_secreted*, *IGHG1_secreted*, *IGHG2_secreted*, *IGHG3_secreted*, and *IGHG4_secreted* (**Figure 10F**). We divided the IgE_≥7_ population into three subpopulations: cells containing *IGHE_secreted* transcripts only, cells containing transcripts of multiple classes of immunoglobulins with a low IRF4 to IR8 ratio, and cells containing transcripts of multiple classes of immunoglobulins with a high IRF4 to IRF8 transcript ratio. We noticed that cells with only *IGHE_secreted* transcripts had a low *IRF4, PRDM1*, and *XBP1* but a high *CD52, HLA-DRA, FOSB, IL4R* and *S100A9* (**Figures 10G, H**). Interestingly, cells in this subcategory had relatively the highest surface expression of alpha-gal-specific immunoglobulins as well as CCR6 (CD196) (**Figure 10G**). Cells with a low ratio of *IRF4* to *IRF8* transcript had the highest expression of MHC class II transcripts, including *HLA-DRA* and *HLA-DPA1, CD37, CD52, CD69, CD74*, and *CD79A*, with an intermediate expression of transcription factors *POU2AF1* and surface CD27 and CD38. Cells with a high ratio of IRF4 to IRF8 transcript contained the highest transcripts of *POU2AF1, PRDM1, XBP1, MZB1*, and *TNRSF17.* In addition, they expressed high surface CD27 and CD38, low CD19 and CD20; all hallmarks of PC/PB (**Figures 10G, H**). The counts of transcripts of multiple Ig classes were highest in IgE_≥7_ subpopulations.

In the current study, samples were collected from participants with AGS at a time when they were actively avoiding allergen. Thus, we are unable to enumerate or characterize the likely present population of non-IgE-expressing type 2-polarized IgG memory B cells that are poised to class switch. Performing a similar analysis in the setting of allergen challenge is a future goal and will allow us to identify and characterize the presence of those cells available to undergo isotype class switching and produce IgE.

## Discussion

A first “inside-out” analysis of single-cell RNA transcriptome data and surface marker expression, we report unique clusters of immune cells in humans with AGS, including mast cell progenitors in the peripheral circulation. The integrated analysis identified several populations that are difficult to detect and correctly identify using flow cytometry or scRNA-seq alone, such as plasmablast, alpha-gal specific IgG1 memory cells, and NKB cells. Overall, we identified 43 distinct functional clusters of immune cells, some of which were differentially contributed by AGS subjects. In AGS subjects, we observed a robust increase in the levels of *S100A9, IFITM3*, and *THBS1* across various clusters, such as CD4 T cells, NK cells, NKT cells, and NKB cells. *S100A9* was also found to be more prevalent in B cells, including PB/PC and neutrophils, while *IFITM3* and *THBS1* were more prevalent in basophils, dendritic cells, and monocytes. Interestingly, *S100A9, IFITM3*, and *THBS1* all possess immunomodulatory activities and play a prominent role in innate immunomodulation and regulation of inflammatory response (34, 52–54). It is, therefore, fair to conclude the immune response to tick bite and subsequent production of alpha-gal-sIgE might be the culmination of the interaction of tick-derived molecules and various immune cells.

We were intrigued by the detection of several clusters of NKT cells, which were largely contributed by the AGS subjects. NKT cells are unique since it has a combination of both innate and effector memory-like functions, and a single cell could produce a larger amount of either Th1, Th2, or Th17 cytokines rapidly following stimulation, providing an efficient effector function (55, 56). In addition, NKT cells interact with multiple immune cells, including B cells, dendritic cells, macrophages, neutrophils, NK cells, γδ T cells, and T cells and modulate their function. NKT and NKT-like cells are of special interest to AGS since we recently reported the presence of glycolipid in the saliva of lone star tick (26). NKT cells can recognize glycolipid antigens with alpha-linked hexose sugars in a context of MHC-like CD1d molecules on B cells and can provide cognate help leading to alpha-gal sIgE production. Interestingly, Liu and colleagues noted that CD1d-mediated interaction between NKT cells and B cells is essential for B cell proliferation since anti-alpha-gal antibody production was inhibited by neutralizing anti-CD1d antibody (57). We were able to detect the CD4^+^-NKT cell cluster (C32) that has been implicated in the Th2 response (58–60). Interestingly, almost all cells (96%) in this cluster were contributed by AGS subjects. Another subset of NKT, CD4^–^NKT cells, is linked to the production of Th1 cytokines but can also produce Th2 cytokines in special situations, thus influencing adaptive immune response toward either type 1 or type 2 immunity during sensitization (58, 59). The mechanisms through which NKT determines the direction of immune responses are not well understood. We observed higher *IL32* levels in NK and NKT clusters in AGS subjects, which has been linked to both pro- and anti-inflammatory responses (61–63). Two clusters of CD4^-^NKT (C19, C22) had upregulated Th1 transcription factors *TBX21* and *PRF1* (Perforin 1), suggesting their cytotoxic nature. In AGS subjects, however, downmodulation occurred for transcripts of *DUSP2* and *PRF1*, associated with inflammatory response and cell killing, respectively, implying inhibition of type 1 immune response (64, 65). In contrast, molecules involved in T-cell and B-cell interaction, such as *CD37* (in C22), and class II MHC functions, such as *CD74, HLA-DPA1*, and *HLA-DRA*, were upregulated, suggestive of transition to type 2 immune responses (66–68). The human immune response panel we used in this experiment lacked CD1d, although other members of the CD1 family, including CD1A and CD1C, that recognize lipids on APC were detected; this contrasts with mice, which only express CD1d molecules (69). The majority of lipid-reactive NKT cells in humans are type II, with diverse use of TCR and TCR repertoire (diverse NKT), while in mice, invariant NKT (iNKT) or type I NKT predominates (70, 71). We did not investigate the nature of the TCR chain in this experiment.

ScRNA-seq library, including both transcriptome and AbSEq-Ab-O, led to the identification of rare NKB cells. NKB cells (C31) possessed a distinct transcriptome compared to NK cells or B cells alone and included signature transcripts of both cell types, including molecules involved in antigen presentation, B cell differentiation into immunoglobulin-secreting cells, and cytolytic activity. The presence of high-level transcripts of *PRF1, GNLY*, and *GZMA* implies these cells have cytolytic properties like NK cells, while the presence of *CD40, CD79A, HLA-DR, MZB1, IgM, and IgD* argues for B cell function. The findings of the presence of CD1 family members *CD1A* and *CD1C* along with *CD79A* and *CD40* in NKB cells were intriguing since they could act as alpha-glycolipid-specific antigen triggers for NKB cells and, therefore, could contribute to the secretion of alpha-gal-specific immunoglobulins. Furthermore, the presence of IgHG1_secreted, although to a low level, suggests the plasticity of NKB cells and a novel switch from innate to adaptive immunity. We did not detect immunoglobulins IgM or IgA in this cluster, as previously reported (72). Since glycolipid antigens have been found in several pathogens, including those found in tick saliva, NKB cells, along with NKT cells, may provide the first line of defense and transition from innate to adaptive immune responses.

The capture of 160,000 B cells (36% of the population) for multiomic analysis allowed the identification of diverse subsets of B cells in 15 clusters, including naïve, transitional, unswitched memory, CD27^+^ memory, CD27^-^ memory, activated B cells, PB/PC and alpha-gal-specific memory B cells. C15 and C16 were activated B cells with upregulated MHC class II, and B cell coreceptor CD79. These clusters also displayed upregulation of transcription factors *IRF4, POU2AFI, PRDM*, and *XBP1* associated with transitioning to plasmablasts/plasma cells (49). It was striking to observe alpha-gal-specific IgG memory B cells in C26 that also exhibited the highest expression of CD196. Within C29, there was a distinct population of switched memory B cells characterized by high levels of CD19, CD20, CD27, CCR6, and CD40. These cells exhibited low levels of IgG transcripts and alpha-gal expression and contained low-level IGHE transcripts. These findings suggest the presence of alpha-gal-specific IgE memory cells within this population.

Since our panel of AbSeq Ab lacked CD138, we were not able to distinguish between PB and PC. The majority of PB/PC (>85%) detected in clusters were contributed by AGS subjects. Interestingly, all six clusters of PB/PC had higher transcripts of *TLR2*, and five of them had higher *TLR4* and *TLR8*. The expression of TLRs on B cells provides a critical link between innate and adaptive immunity: TLR for innate immunity and BCR for adaptive immunity (73). Dual engagement of TLR and B cell receptor (BCR) by PAMPs leads to B cell activation, resulting in IgM and IgA production with limited class switch recombination (CSR) (74). In this context, we observed that PB/PC clusters C30 and C31 primarily contained IGHA_secreted transcripts, while C41 and C42 contained IGHM_secreted transcripts. We, however, also found that C36 and C40 were highly enriched in IGHG1_secreted and IGHG2_secreted transcripts, suggesting CSR did occur. In this regard, there are reports that engagement of TLR with BCR upregulates MHC II and B cell co-receptors CD40 and CD80/86, thereby priming these B cells to receive T cell help that could lead to somatic hypermutation and class switch to IgE production (75–77). In keeping with this, Chandrasekhar and colleagues reported ligands for TLRs, including TLR2 and TLR4 in tick extract, and demonstrated that both MyD88 (through which TLR signals) and B cell co-receptor help are critical for IgE response in their mouse model of AGS (16). In tick saliva-induced models of AGS in zebrafish, the involvement of TLR and Th2 immune response was also noted (78). We have recently shown that infestation of AGKO mice with *A. americanum* nymphs resulted in upregulation of pattern recognition receptors (PRR) of several classes, including *TLR2* and *TLR8*, and sensor and co-receptor for bacterial lipopolysaccharide (LPS) *CD14* and *CD36*, respectively (14). The upregulation of MyD88, which is critical for TLR signaling for cytokine induction, was upregulated as well. Furthermore, we also observed the induction of costimulatory molecules, including *CD40, CD40LG*, and *ICOS*, which could facilitate efficient T-cell help. It is tempting to speculate that engagement of TLR with BCR following tick infestation leads to B cell activation. Further, the presentation of alpha-gal-containing glycolipid by B cells to NKT cells could prime for robust Th2-biased help, leading to CSR and production of alpha-gal-specific IgE.

We relied on transcripts of IGHE_secreted to detect IgE-secreting cells since AbSeq Ab-O against surface IgE is neither available nor could be custom-made. We were able to detect 85 cells that had a minimum of seven copies of IgE transcripts. However, only eight cells contained the IGHE_secreted transcripts exclusively, other cells contained transcripts of various classes of Ig along with IgE. Although the results were surprising to us, there is another study that also employed scRNA-seq and identified two or more V_H_DJ_H_ or V_L_JL recombination patterns in single B cells including naïve B cells, memory B cells, and plasma cells suggesting the existence of novel patterns of Ig gene rearrangement and class switching in a single B cell (79). The cells that largely contained transcripts of IGHE_secreted had low levels of transcription factors *IRF4, POU2AF1, PRDM1*, and *XPB1*, suggesting that they were not plasmablast or plasma cells. These cells expressed higher levels of CD19 and CCR6, were CD27^+^, and had upregulated *CD52, HLA-DRA, FOSB, IL4R*, and *S100A9*, implying that they had encountered antigens and were activated. It was striking to observe that this population was highly recognized by cetuximab, a chimeric antibody containing alpha-gal, which could recognize anti-alpha-gal-specific Ig (80). The expression of high CCR6 on IGHE_secreted cells is consistent with the findings of Cox and colleagues, who noted that alpha-gal sIgE-secreting B cells expressed higher CCR6 (81). It is noteworthy to observe upregulated *S100A9, FOSB*, and *HLA-DRA* in this population. S100A9 acts as an alarmin and binds to PRR, including TLR4, which could induce the Th2 polarization (34).

The upregulation of *CD74* was found in AGS subjects in antigen-presenting cells (APCs) as well as other cell types, independent of MHC class II. The intracellular domain of CD74 functions as a molecular chaperone of MHC II for effective antigen presentation, while the extracellular portion acts as a high-affinity membrane receptor for macrophage migration inhibitory factor (MIF) and d-dopachrome tautomerase (DDT/MIF2) (67, 82, 83). High CD74 expression is found in human carotid plaques and is inversely associated with high-density lipoprotein and statin treatment, suggesting a link to atherosclerosis (84, 85). MIF can trigger the production of a chemokine CXCL8 in T cells, which is also associated with the progression of atherosclerosis (86). Elevated levels of *CXCL8* were observed in several clusters in AGS subjects. Additionally, another chemokine associated with atherosclerosis, *CXCL16*, was found to be elevated in monocyte and basophil clusters (87). Moreover, upregulation of CCL5 was noted in several clusters in AGS subjects, which were also detected in atherosclerotic plaques along with its receptors (88). These findings are significant and could potentially explain the increased risk of coronary artery disease (CAD) in AGS subjects (89).

The exact mechanism of how a tick bite causes human sensitization against alpha-gal and leads to the development of AGS remains poorly understood. Identification and functional characterization of the human immune response in AGS are vital for developing interventions to prevent and manage this unique allergic disease. This is the first detailed immune profiling of circulating cells from participants with AGS and our data will begin to fill the numerous knowledge gaps, and perhaps more importantly, establish that a combined surface marker and transcriptomic approach is possible even in rare allergic diseases Our findings suggest that tick bites may induce a population of circulating mast cell progenitors, and that innate-like cells, such as NKT and NKB cells, play a critical role in sensitization to alpha-gal. Furthermore, alpha-gal-specific IgE is secreted by a heterogeneous population of B cells, including CCR6-proficient B cells and CCR6-deficient plasmablast/plasma cells. Future applications of these findings and approach will broaden our understanding of IgE responses in general, the potential relationship between an allergic condition and atherosclerosis, as well as the role of ectoparasites in shaping Th2-related immune responses in a modern world. Furthermore, studies aim to elucidate specific populations that respond to relevant tick antigens as we look to develop an intervention for this expanding allergic condition.

## Data Availability

The original contributions presented in the study are publicly available. This data can be found here: https://www.ncbi.nlm.nih.gov/geo/query/acc.cgi?acc=GSE308814.
