# Supplementary material for: Single-cell mRNA analysis and surface marker expression profiling of circulating immune cells in humans with alpha-gal syndrome
Source: Front Immunol. 2025 Sep 30;16:1629310. doi: 10.3389/fimmu.2025.1629310 (PMC12518106; doi:10.3389/fimmu.2025.1629310)
Supplement: Supplementary file 1 [file DataSheet1.pdf]

Table S1. BD Rhapsody Immune Response Panel Hs (Gene Symbol from DOC ID 55665, Rev. 2.0)

ADA	CCR1	CD72	CXCR6	GZMH	IL1RL1	LAMP3	POU2AF1	TMEM97
ADGRE1	CCR10	CD74	DEFA3	GZMK	IL1RN	LAP3	PRDM1	TNF
ADGRG3	CCR2	CD79A	DEFA4	HAVCR2	IL2	LAT	PRF1	TNFRSF13C
AIM2	CCR3	CD79B	DOCK8	HLA-A	IL21	LAT2	PSEN1	TNFRSF17
ALAS2	CCR4	CD80	DPP4	HLA-DMA	IL22	LCK	PTGDR2	TNFRSF25
ANXA5	CCR5	CD86	DUSP1	HLA-DPA1	IL23R	LEF1	PTPRC	TNFRSF4
AOC3	CCR7	CD8A	DUSP2	HLA-DQB1	IL25	LGALS1	PTTG2	TNFRSF8
APOBEC3G	CCR8	CD8B	DUSP4	HLA-DRA	IL2RA	LGALS3	QPCT	TNFRSF9
APOE	CCR9	CD9	EBF1	HMMR	IL2RB	LGALS9	RGS1	TNFSF10
AQP9	CD14	CEACAM8	EGR1	ICAM1	IL3	LIF	RNASE2	TNFSF13
ARG1	CD160	CHI3L1	EGR3	ICOS	IL31	LILRB4	RNASE6	TNFSF13B
ARL4C	CD163	CHI3L2	ELANE	IER3	IL32	LIPA	RORA	TNFSF14
ATF6B	CD1A	CLC	ENTPD1	IFITM2	IL33	LRRC32	RORC	TNFSF8
AURKB	CD1B	CLEC10A	EOMES	IFITM3	IL3RA	LTA	RPN2	TOP2A
AZU1	CD1C	CLEC4D	EPX	IFNA1	IL4	LTB	RUNX3	TPSAB1
B3GAT1	CD2	CLEC4E	F13A1	IFNG	IL4R	LY86	S100A10	TRAC
BACH2	CD200	CMKLR1	F5	IFNGR1	IL5	LYN	S100A12	TRAT1
BAX	CD209	CMTM2	FAM129C	IGBP1	IL6	MCM2	S100A9	TRBC2
BCL11B	CD22	CNOT2	FAM65B	IGHA1<br>_secreted	IL7R	MCM4	SELL	TRDC
BCL2	CD24	CNTNAP3	FAS	IGHD<br>_membrane	IL9	MGST1	SELPLG	TREM1
BCL2A1	CD244	CPA3	FASLG	IGHE<br>_secreted	IRF4	MITF	SLC25A37	TRIB2
BCL6	CD247	CR2	FCER1A	IGHG1<br>_membrane	IRF8	MKI67	SLC7A7	TSPAN32
BIN2	CD27	CSF2	FCER1G	IGHG1<br>_secreted	ITGA4	MME	SNCA	TXK
BIRC3	CD274	CSF3	FCER2	IGHG2<br>_secreted	ITGAE	MMP12	SPP1	TYMS
BLK	CD28	CST7	FCGR3A	IGHG3<br>_secreted	ITGAM	MMP9	STAT1	UBE2C
BLNK	CD300A	CTLA4	FCN1	IGHG4<br>_secreted	ITGAX	MS4A1	STAT3	VEGFA
BPI	CD33	CTSD	FLT3	IGHM<br>_membrane	ITGB2	MYC	STAT4	VMO1
BTG1	CD34	CTSG	FN1	IGHM<br>_secreted	JCHAIN	MZB1	STAT5A	VNN2
BTLA	CD36	CTSW	FOSB	IGKC	JUN	NAMPT	STAT6	VPREB3
C10orf54	CD37	CX3CR1	FOSL1	IGLC3	JUNB	NCAM1	TARP<br>_refseq	VPS28
C1QA	CD38	CXCL1	FOXO1	IKZF1	KCNE3	NCR3	TBX21	VSIG4
C1QB	CD3D	CXCL10	FOXP1	IKZF2	KDELR1	NINJ2	TCF4	XBP1
CASP5	CD3E	CXCL11	FOXP3	IL12A	KIAA0101	NKG7	TCF7	YBX3
CBLB	CD3G	CXCL13	FTH1	IL12RB1	KIR2DL1	NRP1	TCL1A	ZAP70
CCL1	CD4	CXCL16	FUT4	IL12RB2	KIT	NT5E	TGFB1	ZBED2
CCL13	CD40	CXCL2	FYB	IL13	KLRB1	PASK	TGFB3	ZBTB16
CCL17	CD44	CXCL3	FYN	IL15	KLRC1	PAX5	TGFBI	ZNF683
CCL19	CD48	CXCL5	GAB2	IL15RA	KLRC3	PCNA	THBD	
CCL2	CD5	CXCL8	GAPDH	IL17A	KLRC4	PDCD1	THBS1	
CCL20	CD52	CXCL9	GIMAP2	IL17F	KLRF1	PDIA4	TIAF1	
CCL22	CD6	CXCR1	GIMAP5	IL18	KLRG1	PDIA6	TIGIT	
CCL3	CD63	CXCR2	GNAI2	IL18R1	KLRK1	PI3	TLR2	
CCL4	CD69	CXCR3	GNLY	IL18RAP	LAG3	PIK3AP1	TLR7	
CCL5	CD7	CXCR4	GZMA	IL1B	LAIR2	PIK3IP1	TLR8	
CCND2	CD70	CXCR5	GZMB	IL1R2	LAMP1	PMCH	TLR9	

Table S2. Additional TrueSeq combinatorial dual index primers

Primers	Sequence
TruSeq D502	AATGATACGGCGACCACCGAGATCTACACATAGAGGCACACTCTTTCCC TACACGACGCTCTTCCGAT*C*T
TruSeq D503	AATGATACGGCGACCACCGAGATCTACACCTATCCTACACTCTTTCCCT ACACGACGCTCTTCCGAT*C*T
TruSeq D701	CAAGCAGAAGACGGCATACGAGATCGAGTAATGTGACTGGAGTTCAGAC GTGTGCTCTTCCGATC*T
TruSeq D702	CAAGCAGAAGACGGCATACGAGATTCTCCGGAGTGACTGGAGTTCAGAC GTGTGCTCTTCCGATC*T
TruSeq D703	CAAGCAGAAGACGGCATACGAGATAATGAGCGGTGACTGGAGTTCAGAC GTGTGCTCTTCCGATC*T
TruSeq D704	CAAGCAGAAGACGGCATACGAGATGGAATCTCGTGACTGGAGTTCAGAC GTGTGCTCTTCCGATC*T
TruSeq D705	CAAGCAGAAGACGGCATACGAGATTTCTGAATGTGACTGGAGTTCAGAC GTGTGCTCTTCCGATC*T
TruSeq D706	CAAGCAGAAGACGGCATACGAGATACGAATTCGTGACTGGAGTTCAGAC GTGTGCTCTTCCGATC*T
TruSeq D707	CAAGCAGAAGACGGCATACGAGATAGCTTCAGGTGACTGGAGTTCAGAC GTGTGCTCTTCCGATC*T
TruSeq D708	CAAGCAGAAGACGGCATACGAGATGCGCATTAGTGACTGGAGTTCAGAC GTGTGCTCTTCCGATC*T
TruSeq D709	CAAGCAGAAGACGGCATACGAGATCATAGCCGGTGACTGGAGTTCAGAC GTGTGCTCTTCCGATC*T
TruSeq D710	CAAGCAGAAGACGGCATACGAGATTTCGCGGAGTGACTGGAGTTCAGAC GTGTGCTCTTCCGATC*T
TruSeq D711	CAAGCAGAAGACGGCATACGAGATGCGCGAGAGTGACTGGAGTTCAGA CGTGTGCTCTTCCGATC*T
TruSeq D712	CAAGCAGAAGACGGCATACGAGATCTATCGCTGTGACTGGAGTTCAGAC GTGTGCTCTTCCGATC*T

* 3' Phosphorothioate bond(s)

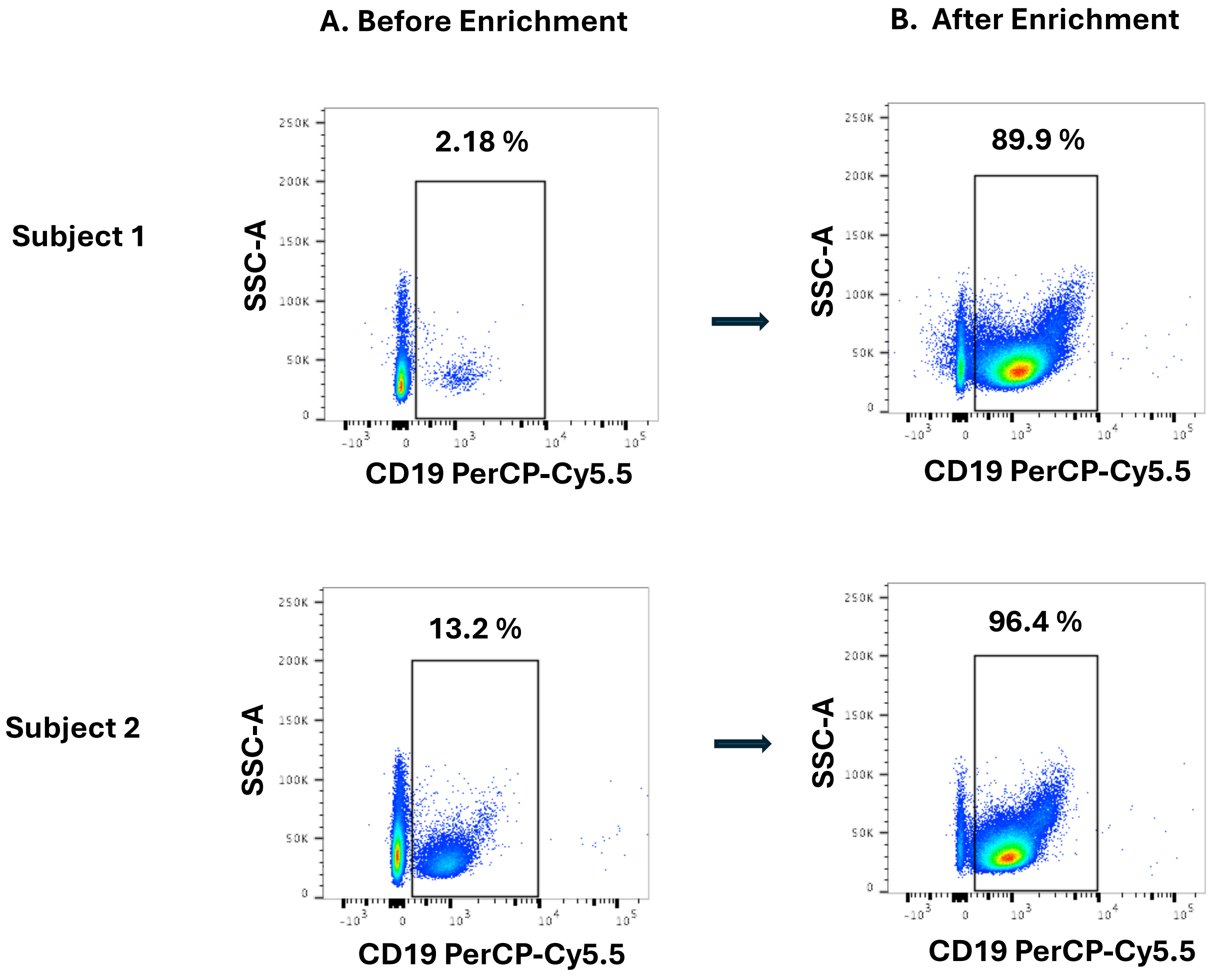

Figure S1. Enrichment of B cells from peripheral blood mononuclear cells. Flow cytometric analysis shows the percentage of B cells before enrichment (A) and after enrichment (B), involving negative selection, in a representative experiment.

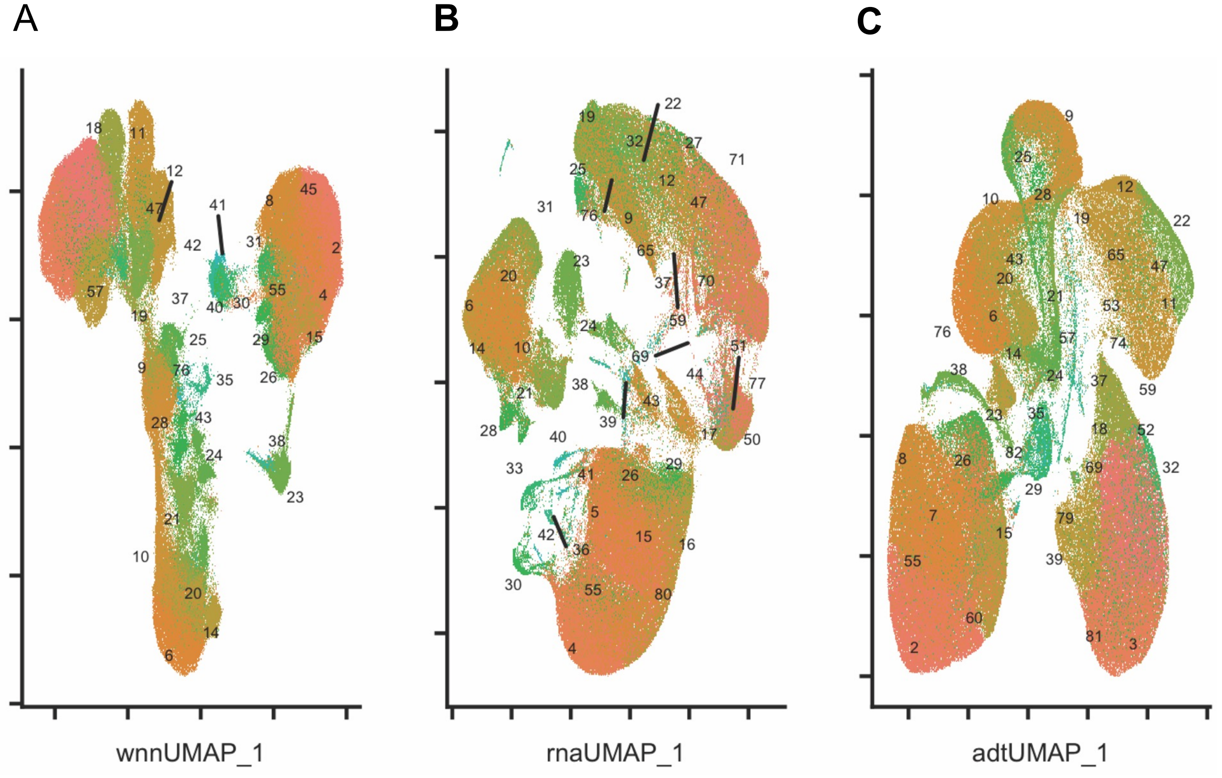

Figure S2. Analysis of the BD Rhapsody dataset using weighted-nearest neighbor (WNN) multimodal single-cell analysis. UMAP visualizations are computed using WNN (**A**), RNA (**B**), and protein analysis (**C**).

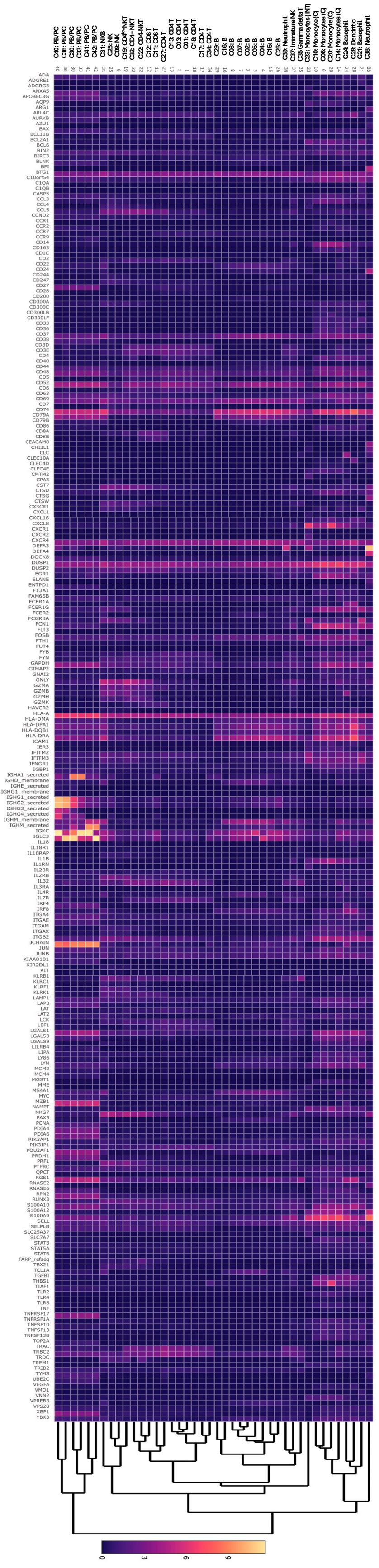

Figure S3. Major cell populations identified in WNN clusters. Heatmap showing transcriptomes in WNN clusters.

Table S3. Distribution of cells in each WNN cluster

Clusters	Cell type	AGS cell counts (%)	Control cell counts (%)
Total cells	All Cells	284078 (64.9)	153692 (35.10)
C1	CD4 T cells	20757 (70.36)	8744 (29.64)
C2	B cells	17329 (68.41)	8003 (31.59)
C3	CD4 T cells	15773 (66.12)	8083 (33.38)
C4	B cells	12892 (59.20)	8884 (40.80)
C5	B cells	12143 (56.77)	9247 (43.23)
C6	Monocytes	12307 (59.19)	8487 (40.81)
C7	B cells	11669 (59.47)	7954 (40.53)
C8	B cells	8279 (43.23)	10848 (56.72)
C9	NK cells	10475 (55.51)	8394 (44.49)
C10	Monocytes	10620 (69.44)	4673 (30.56)
C11	CD8 T cells	8045 (57.50)	5947 (42.50)
C12	CD8 T cells	6737 (51.82)	6265 (48.18)
C13	CD4 T cells	8181 (63.63)	4677 (36.37)
C14	Monocytes	10458 (82.33)	2244 (17.67)
C15	B cells	6364 (50.42)	6257 (49.58)
C16	B cells	7967 (63.96)	4489 (36.04)
C17	CD4 T cells	6051 (57.47)	4478 (42.53)
C18	CD4 T cells	1666 (18.47)	7352 (81.53)
C19	$CD4^-$ NKT cells	6860 (77.94)	1942 (22.06)
C20	Monocytes	7171 (83.92)	1384 (16.18)
C21	Basophils	6224 (72.89)	2315 (27.11)
C22	$CD4^-$ NKT cells	5458 (70.60)	2273 (29.40)
C23	Monocytes	7536 (97.69)	178 (2.31)
C24	Basophils	4491 (63.12)	2624 (36.88)
C25	NK cells	4841 (74.66)	1643 (25.34)
C26	B cells	3220 (50.03)	3216 (49.97)
C27	$CD4^+CD8^+$ T cells	3157 (67.23)	1539 (32.77)
C28	Dendritic cells	2557 (69.20)	1138 (30.80)
C29	B cells	3100 (95.95)	131 (4.05)
C30	Plasmablast/Plasma Cells	2230 (89.27)	268 (10.73)
C31	NKB cells	2084 (84.44)	384 (15.56)
C32	$CD4^+$ NKT cells	1894 (95.90)	81 (4.10)
C33	Plasmablast/Plasma Cells	1592 (83.97)	304 (16.03)
C34	CD4 T cells	1607 (90.33)	172 (9.67)
C35	Gamma delta T Cells	822 (54.44)	688 (45.56)
C36	Plasmablast/Plasma Cells	1024 (88.58)	132 (11.42)
C37	Immature NK cells	479 (43.90)	612 (56.10)
C38	Neutrophils	675 (83.23)	136 (16.77)
C39	Neutrophils	626 (82.48)	133 (17.52)
C40	Plasmablast/Plasma Cells	463 (76.28)	144 (23.72)
C41	Plasmablast/Plasma Cells	263 (86.23)	42 (13.77)
C42	Plasmablast/Plasma Cells	191 (85.65)	32 (14.35)
C43	Mast cells	158 (88.76)	20 (11.24)

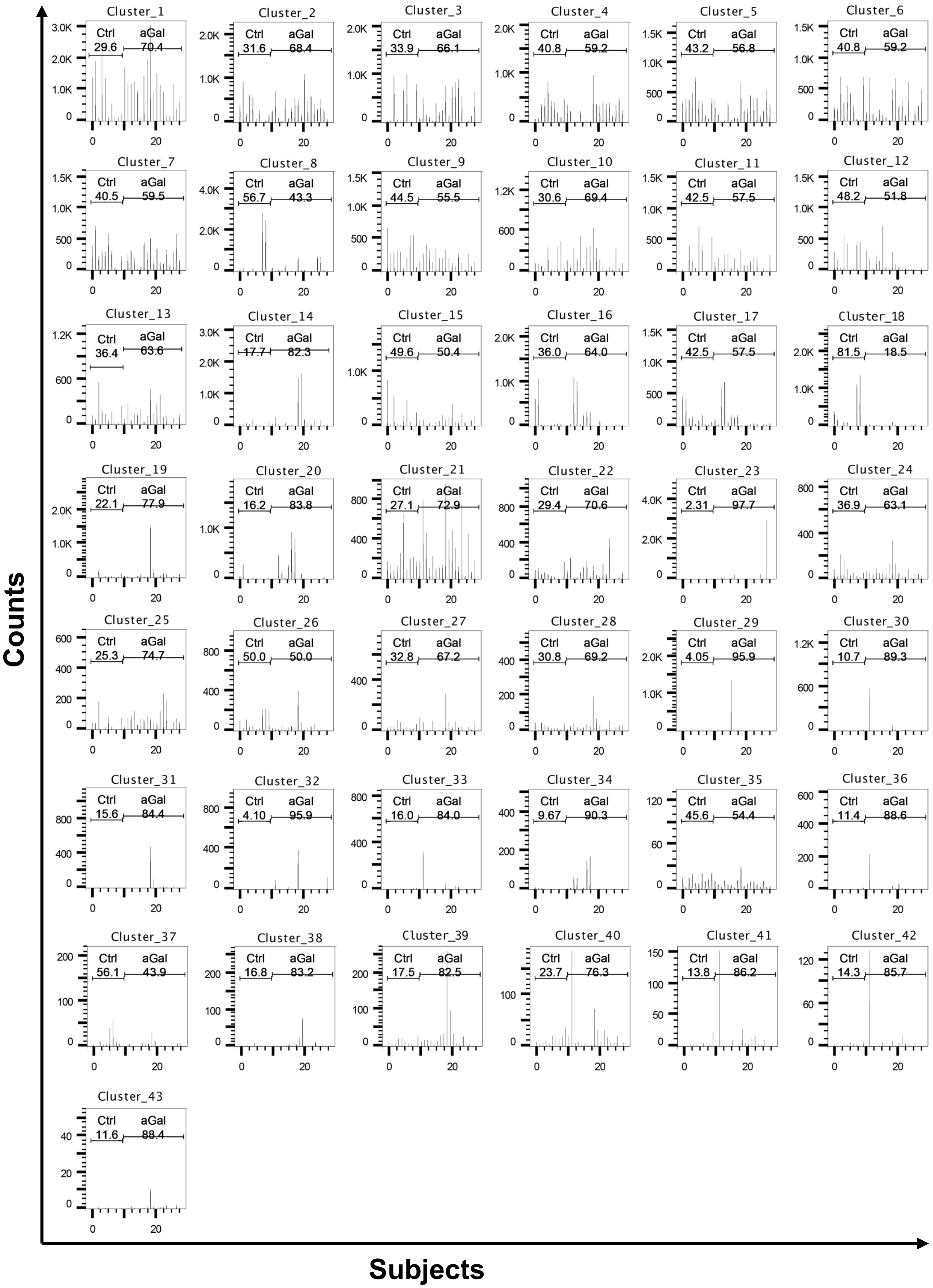

Figure S4. Contribution of cells from subjects in WNN clusters. The order of subjects in each plot for control subjects (Ctrl) is UNC0-240, 239, 233, 229, 142, 095, 039, 029, 026, 216, and for AGS subjects (aGal) is UNC0-234, 222, 237, 236, 235, 232, 231, 230, 225, 224, 221, 219, 218, 217, 210, 209, 212, and 211.

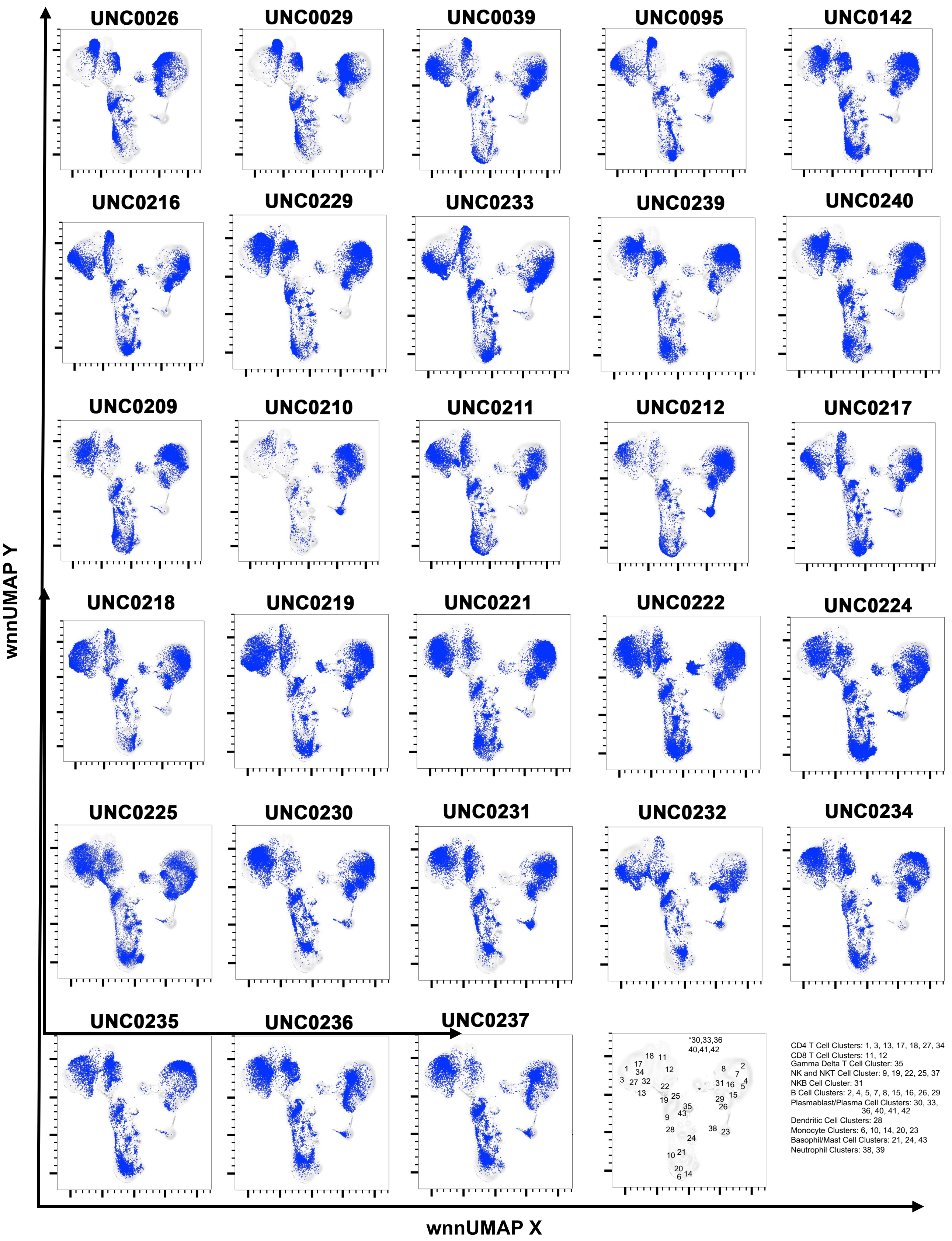

Figure S5. Distribution of WNN clusters across individual subjects.
